# Efficacy and safety of distal transradial access for coronary angiography and percutaneous coronary intervention: a meta-analysis

**DOI:** 10.3389/fcvm.2025.1530995

**Published:** 2025-03-18

**Authors:** Qinyan Yang, Xianli Wei, Jianyu Wu, Chunlan Li, Yuechen Qin, Haijian Zeng, Mengtian Qin, Yue Zou, Shiming Zhang, Weiming Liang, Jie Li

**Affiliations:** The First Affiliated Hospital of Guangxi University of Science and Technology, Guangxi University of Science and Technology, Liuzhou, Guangxi, China

**Keywords:** coronary artery disease, coronary angiography, percutaneous coronary intervention, transradial access, radial artery occlusion, distal transradial access, meta-analysis

## Abstract

**Introduction:**

This meta-analysis aims to evaluate the efficacy and safety of dTRA for coronary angiography (CAG) and percutaneous coronary intervention (PCI) in comparison to cTRA.

**Materials and methods:**

Four databases (PubMed, Embase, Web of Science, and Cochrane Library) were searched from their inception to 13 April 2024 for studies comparing dTRA and cTRA in coronary diagnostic or interventional catheterization. The meta-analysis evaluated radial artery occlusion (RAO), procedure success, the success rate of catheter puncture, the success rate of a single attempt, hematoma occurrence, radial artery spasms, puncture site bleeding, puncture time, procedural time, the dosage of contrast medium, and hemostasis time.

**Results:**

A total of 31 studies were included in the meta-analysis. Compared with cTRA, dTRA significantly reduced the incidence of RAO [odds ratio (OR) = 0.41, 95% CI: 0.34–0.50, *P* < 0.05], hematoma (OR = 0.67, 95% CI:0.56–0.80, *P* < 0.05), and shorter hemostasis time [weighted mean difference (WMD) = −0.43, 95% CI:−0.65 to −0.20, *P* < 0.05] but had a significantly lower procedure success rate (OR = 0.41, 95% CI: 0.30–0.56, *P* < 0.05), a lower catheter puncture success rate (OR = 0.44, 95% CI: 0.27–0.71, *P* < 0.05), and a longer puncture time (WMD = 0.60, 95% CI: 0.44–0.75, *P* < 0.05). No significant differences were observed between dTRA and cTRA in terms of the success rate of a single attempt, radial artery spasms, puncture site bleeding, procedural time, and dosage of contrast medium.

**Conclusions:**

Our results revealed that dTRA is a workable and safe method for cardiovascular interventional diagnostics and treatment. It significantly reduces the incidence of RAO and hematoma, as well as shortens hemostasis time following surgery.

**Systematic Review Registration:**

https://www.crd.york.ac.uk/prospero/display_record.php?ID=CRD42024596238, PROSPERO (CRD42024596238).

## 1 Introduction

Coronary artery disease (CAD) remains one of the leading causes of mortality, as reported by the World Health Organization (1). Coronary angiography (CAG) and percutaneous coronary intervention (PCI) are essential procedures for the diagnosis and treatment of CAD. These procedures can be performed using transradial, transbrachial, or transfemoral access. Just 12 years later, in 1989, Campeau reported the first 100 diagnostic CAGs using the radial approach, following the first successful femoral artery PCI in 1977 (2). The RIVAL study later verified the safety and high success rate of this approach, establishing it as the preferred referral technique for catheterization in compliance with the guidelines of the European Society of Cardiology (3).

Unfortunately, this procedure carries certain risks, including bleeding, hematoma, and pseudoaneurysm. In addition, prolonged bed relaxation following the procedure raises the risk of lower limb thrombosis and pulmonary embolism (4). Transradial artery access (TRA) is associated with fewer bleeding and vascular complications and significant safety advantages over transfemoral artery access (5). In 1993, Kiemeneij and Laarman, respectively, showed that TRA performed better than transfemoral access in a variety of areas, such as a lower risk of bleeding, a shorter length of hospital stay, early mobilization, reduced costs, and higher patient satisfaction; as a result, TRA has become the preferred approach for CAG (6–9). Despite its benefits, TRA also has certain drawbacks. These drawbacks include mild bleeding at the access site and radial artery occlusion (RAO) after the procedure. RAO, both early and late, which varies in frequency from 5% to 30% in various studies, is the most common complication (5, 10–12).

Babunashvili and Dundua recently developed dTRA, a retrograde method to unblock obstructed ipsilateral radial arteries (13). Its superiority over cTRA lies in providing enhanced comfort for both the patient and the operator throughout the process, and it can be performed without a palpable radial artery in an anatomical snuffbox (14). Moreover, it preserves antegrade blood flow via the superficial palmar arch in instances of RAO. Additional benefits include fewer complications at the puncture site, a shorter hospital stay, and the preservation of the radial artery for revascularization procedures such as coronary artery bypass grafts or the creation of AV-fistulas in patients with chronic kidney disease (15, 16) because these patients often exhibit more complex coronary artery disease and an elevated risk of periprocedural and postprocedural complications, including hemorrhage, thrombotic incidents, and contrast-induced acute kidney damage (17). Having said that, this method is not without its share of disadvantages, some of which include greater difficulty in needling, an increased risk of nerve irritation, and an inability to pass through bigger sheaths (16). Thus, the application of dTRA in interventional surgery for coronary heart disease remains debatable, necessitating more evidence of its safety and efficacy.

Thus, we conducted a meta-analysis to compare the efficacy and safety of dTRA compared to cTRA for coronary angiography and percutaneous coronary intervention.

## 2 Materials and methods

### 2.1 Search strategy

This meta-analysis adhered to the 2020 standards of the Preferred Reporting Items for Systematic Reviews and Meta-Analyses (PRISMA). The research was formally registered with PROSPERO, under registration number CRD42024596238. A comprehensive search was performed across four databases, PubMed, Web of Science, Embase, and the Cochrane Library, to collect relevant studies published until 13 April 2024. The search strategy adhered to the PICOS concept and utilized a combination of MeSH keywords and unconstrained textual phrases. The search method involved combining the terms “coronary disease,” “coronary angiography,” “percutaneous coronary intervention,” “radial artery,” “transradial,” “snuff box,” and “distal radial artery.” Supplementary Tables S1–S4 provide the details of the searched record across the four databases.

### 2.2 Inclusion and exclusion criteria

Inclusion criteria are as follows: (1) patients aged >18 years with an indication for CAG or PCI; (2) patients in the intervention group received dTRA; (3) patients in the control group received cTRA; (4) studies reporting at least one of the following outcomes: RAO, procedure success, success rate of catheter puncture, success rate of a single attempt, hematoma incidence, radial artery spasms, puncture site bleeding, puncture time, procedural time, dosage of contrast medium, or hemostasis time; and (5) study design: randomized controlled trial (RCT), prospective study, or retrospective study.

The exclusion criteria are as follows: (1) studies that were of other types, such as case reports, protocols, letters, editorials, comments, reviews, and meta-analyses; (2) studies that were not relevant; (3) studies that did not compare dTRA vs. cTRA; (4) studies with duplicate patient cohorts; (5) studies where data cannot be extracted; and (6) studies that did not report relative outcomes.

### 2.3 Selection of studies

The literature selection procedure, which entailed eliminating duplicate items, was executed using EndNote (version 20; Clarivate Analytics). Two independent reviewers conducted the preliminary search. Duplicate entries were removed, and the titles and abstracts were evaluated for relevance. Each study was subsequently classified as either included or excluded. We addressed the issue by achieving a consensus. Should the parties fail to reach an agreement, a third reviewer acted as a mediator.

### 2.4 Data extraction

Data extraction was performed independently by two reviewers. The data collected comprised: (1) essential features of the included studies: author, nationality, year of publication, and research design; (2) baseline features of study participants: sample size, male-to-female ratio, age, prevalence of hypertension and diabetes, and smoking habit; (3) primary outcome: incidence of RAO; and (4) secondary outcomes: procedure success, success rate of catheter puncture, success rate of a single attempt, hematoma incidence, radial artery spasm, puncture site bleeding, puncture time, procedural time, dosage of contrast medium, and hemostasis time.

### 2.5 Quality assessment

Two independent reviewers assessed the quality of the included studies. We utilized the Newcastle–Ottawa Scale (NOS) (18) to assess the quality of both retrospective and prospective studies in this analysis, covering eight domains: (1) representativeness of the exposed cohort; (2) selection of the non-exposed cohort; (3) ascertainment of exposure; (4) demonstration that the outcome of interest was not present at the start of the study; (5) comparability of cohorts based on study design or analysis; (6) assessment of the outcome; (7) adequacy of follow-up duration for outcomes to occur; and (8) adequacy of follow-up of cohorts. We evaluated the RCTs using the Cochrane Risk of Bias tool, which encompasses seven domains: (1) random sequence generation; (2) allocation concealment; (3) participant and personnel blinding; (4) outcome assessment blinding; (5) incomplete outcome data; (6) selective reporting; and (7) other biases. We engaged in collaborative deliberation to resolve any inconsistencies in the contested findings.

### 2.6 Statistical analysis

We conducted the study selection process, using EndNote (version 20; Clarivate Analytics) to eliminate duplicates. We utilized Review Manager 5.4 (Cochrane Collaboration, Oxford, UK) to examine the outcomes of all included studies. We analyzed binary variables using odds ratios (ORs) with a 95% confidence interval (CI). We employed the weighted mean difference (WMD) with a 95% CI to analyze continuous variables. We transformed the medians and interquartile ranges of continuous data into means and standard deviations. We evaluated the statistical heterogeneity among the studies used in the analysis using the Cochrane *Q*-test and the *I*^2^ index. An *I*^2^ score greater than 50% signifies a considerable degree of heterogeneity. The random effects model was employed when substantial variability existed among the studies, whereas the fixed effects model was utilized in its absence. Statistical heterogeneity was evaluated using a conventional chi-square test and deemed significant at *P* < 0.05. We assessed the possibility of publication bias by a visual examination of the funnel plots.

## 3 Results

### 3.1 Search results

**Figure 1:**
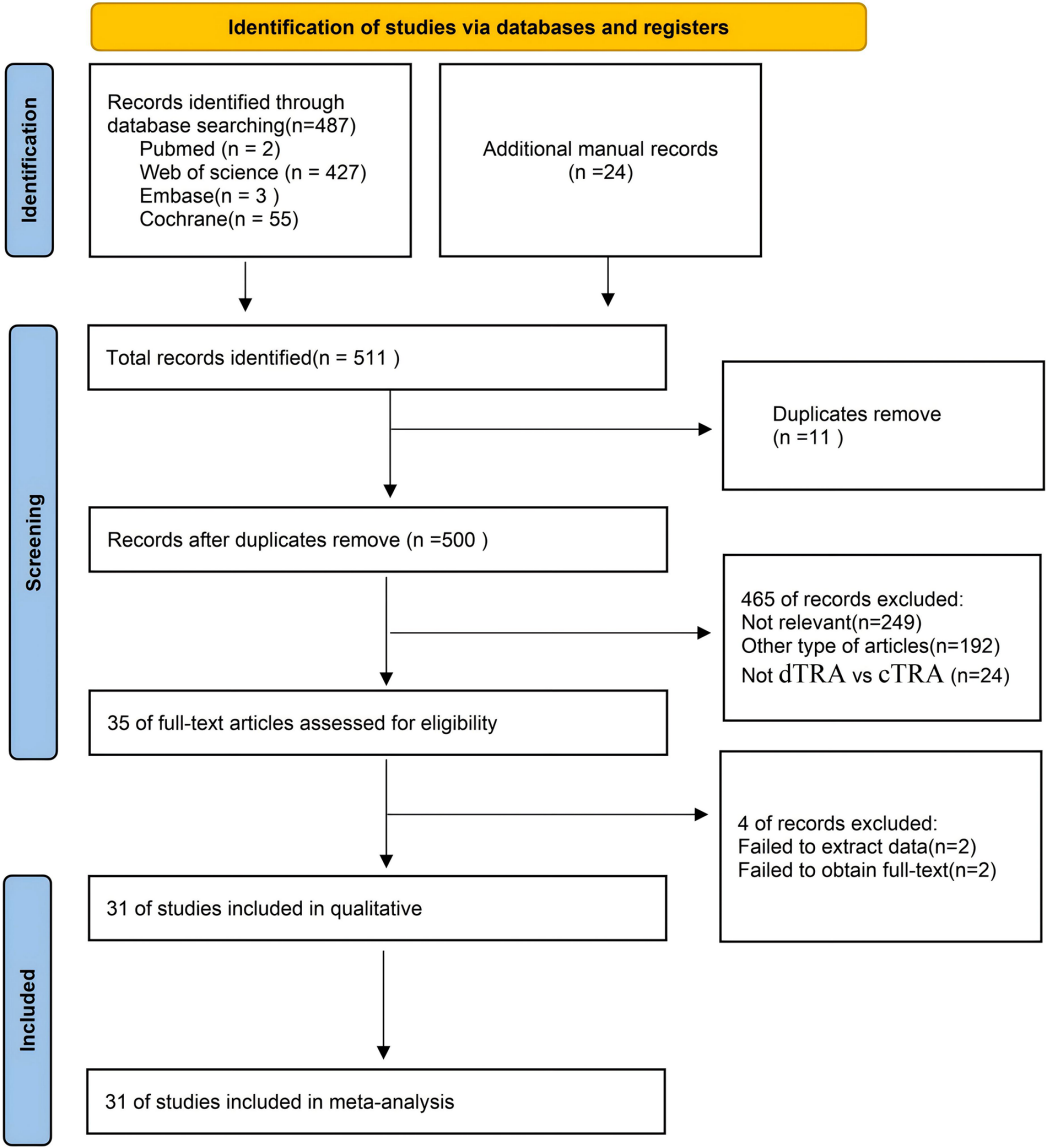
Flowchart of literature search strategies.

Figure 1 illustrates the process of literature selection and inclusion. We acquired a total of 487 publications from four databases and identified an additional 24 articles by examining the bibliographies of the aforementioned studies. We incorporated 31 articles (19–33) in the final meta-analysis, conforming to the predetermined inclusion and exclusion criteria. Figure 1 illustrates the process of choosing and incorporating the literature.

### 3.2 Study characteristics

The meta-analysis comprised 31 studies, consisting of 13 RCTs, 15 prospective studies, and 3 retrospective studies. The meta-analysis comprised a total of 16,891 individuals, including 8,260 patients in the dTRA group and 8,631 patients in the TRA group. The included studies were conducted in several countries, including China (22, 29–31, 34–36), India (19, 21, 37), Russia (20, 38, 39), Turkey (23, 32, 40), Iran (24), Mexico (25), Greece (26, 33), Ireland (27), America (28, 41, 42), Bangladesh (43), Tunisia (44), Nepal (45), Belgium (46), Romania (47), Italy (48), and Egypt (49). Table 1 provides the characteristics of the included studies and patients.

**Table 1:** Characteristics of the included studies.

Study	Country	Design	Group	Cases	Men (%)	Age (mean ± SD)	HT (%)	DM (%)	Smoke (%)
Bhambhani et al. (19)	India	P	dTRA	100	84	54.6 ± 9.2	59	40	39
			cTRA	100	73	54.9 ± 9.7	50	35	32
Korotkikh et al. (20)	Russia	RCT	dTRA	371	65.2	63 ± 10.4	86.8	27.5	29.6
			cTRA	382	66.5	62.6 ± 9.6	86.6	26.7	31.2
Sharma et al. (21)	India	RCT	dTRA	485	60	55 ± 6	NA	NA	NA
			cTRA	485	59	55 ± 7	NA	NA	NA
Chen et al. (22)	China	RCT	dTRA	398	56	65.2 ± 11.9	65.8	18.8	27.4
			cTRA	403	56.3	65.9 ± 11.1	62.5	20.3	31.8
Soydan et al. (23)	Turkey	P	dTRA	27	63	57.6 ± 12.5	77.8	40.7	33.3
			cTRA	43	72.1	60.3 ± 10.8	76.7	39.5	27.9
Roghani-Dehkordi et al. (24)	Iran	P	dTRA	70	62.8	55.1 ± 9.7	57.1	74.2	67.1
			cTRA	56	64.3	56.5 ± 9.6	67.8	80.3	78.6
Eid-Lidt et al. (25)	Mexico	RCT	dTRA	140	75	63.1 ± 10.3	60	51.4	20.4
			cTRA	142	76.7	61.1 ± 11.1	61.9	43.7	16.9
Tsigkas et al. (26)	Greece	RCT	dTRA	518	76.3	65.6 ± 11.1	62.7	29.4	32.6
			cTRA	524	75.6	65.4 ± 12.2	55.4	30.2	32.3
Coughlan et al. (27)	Ireland	P	dTRA	47	83	61 ± 10.17	NA	NA	NA
			cTRA	47	74.5	61.8 ± 10.9	NA	NA	NA
Al-Azizi et al. (28)	USA	RCT	dTRA	150	79.3	65.9 ± 8.7	74.7	34	NA
			cTRA	150	71.3	67.3 ± 10.5	80	30	NA
Li et al. (29)	China	P	dTRA	342	61.7	65.4 ± 10.4	71.6	26.6	40.6
			cTRA	342	60.8	66.2 ± 10.4	71.1	26.9	36.3
Lin et al. (30)	China	P	dTRA	450	45.5	55.3 ± 11.0	24.9	10.7	27.6
			cTRA	450	50	58.8 ± 9.4	25.1	12.4	22.4
Lu et al. (31)	China	P	dTRA	40	57.5	54.3 ± 14.5	NA	15	NA
			cTRA	40	62.5	56.4 ± 13.7	NA	17.5	NA
Kis and Soydan (32)	Turkey	P	dTRA	17	70.6	55.3 ± 14.0	70.6	47.1	35.3
			cTRA	24	70.8	58.4 ± 22.6	79.2	33.3	29.2
Koutouzis et al. (33)	Greece	RCT	dTRA	100	74	63.8 ± 10.9	73	27	35
			cTRA	100	77	62.8 ± 11.0	63	28	28
Amin et al. (43)	Bangladesh	P	dTRA	50	NA	NA	NA	NA	NA
			cTRA	50	NA	NA	NA	NA	NA
Hammami et al. (44)	Tunisia	P	dTRA	82	75	59.2 ± 11.5	40	45	41.5
			cTRA	95	73	60.4 ± 11.9	44	40	42
Gajurel et al. (45)	Nepal	P	dTRA	82	58.5	57.7 ± 10	35.3	25.6	43.9
			cTRA	82	53.6.	57.2 ± 10	29.2	18.2	32.9
Aoi et al. (41)	USA	R	dTRA	202	64.9	69.2 ± 10.2	85.6	37.6	33.3
			cTRA	206	62.6	68.8 ± 10.0	95.1	45.6	25.2
Vefali and Saricam (40)	Turkey	RCT	dTRA	102	70.6	60.9 ± 10.8	54.9	36.2	27.5
			cTRA	103	68	59.8 ± 8.5	53.4	37.8	25.2
Wang et al. (34)	China	P	dTRA	312	51.3	50.1 ± 7.2	60.6	31.4	56.4
			cTRA	308	60	51.2 ± 7.3	56.5	28.2	54.5
Xu et al. (35)	China	R	dTRA	151	70.9	60.1 ± 9.9	62.3	35.8	34.4
			cTRA	151	62.9	60.4 ± 10.6	64.9	36.4	33.1
Aminian et al. (46)	Belgium	RCT	dTRA	650	73.7	68.0 ± 10.7	76.7	30.2	22.4
			cTRA	657	71.2	68.2 ± 11.1	79.6	28.9	21.4
Chugh et al. (42)	USA	R	dTRA	263	70	55.1 ± 11.9	12.1	14	59.3
			cTRA	282	70.3	53.8 ± 12.9	12	13.1	70.9
Koledinskiy et al. (38)	Russia	RCT	dTRA	132	NA	NA	NA	NA	NA
			cTRA	132	NA	NA	NA	NA	NA
Mokbel et al. (47)	Romania	RCT	dTRA	57	NA	NA	NA	NA	NA
			cTRA	57	NA	NA	NA	NA	NA
Kaledin et al. (39)	Russia	P	dTRA	2775	NA	NA	NA	NA	NA
			cTRA	3,099	NA	NA	NA	NA	NA
Lucreziotti et al. (48)	Italy	RCT	dTRA	100	59	71.8 ± 11.4	83	30	50
			cTRA	104	68.3	71.7 ± 10.8	75	28.8	53.8
Feng et al. (36)	China	P	dTRA	527	73.8	65.8 ± 16.7	81	39.1	59.8
			cTRA	586	72.2	66.2 ± 18.2	83	36.5	62.1
Mohamed (49)	Egypt	RCT	dTRA	50	86	56.34 ± 6.08	74	50	54
			cTRA	50	80	57.56 ± 5.49	86	54	48
Lalani et al. (37)	India	P	dTRA	66	59.1	56.0 ± 10.7	56.1	37.9	NA
			cTRA	64	59.4	58.2 ± 12.3	62.5	48.4	NA

R, retrospective study; P, prospective study; RCT, randomized control trial; dTRA, distal transradial approach; cTRA, conventional transradial approach; NA, not available.

### 3.3 Quality assessment

**Figure 2:**
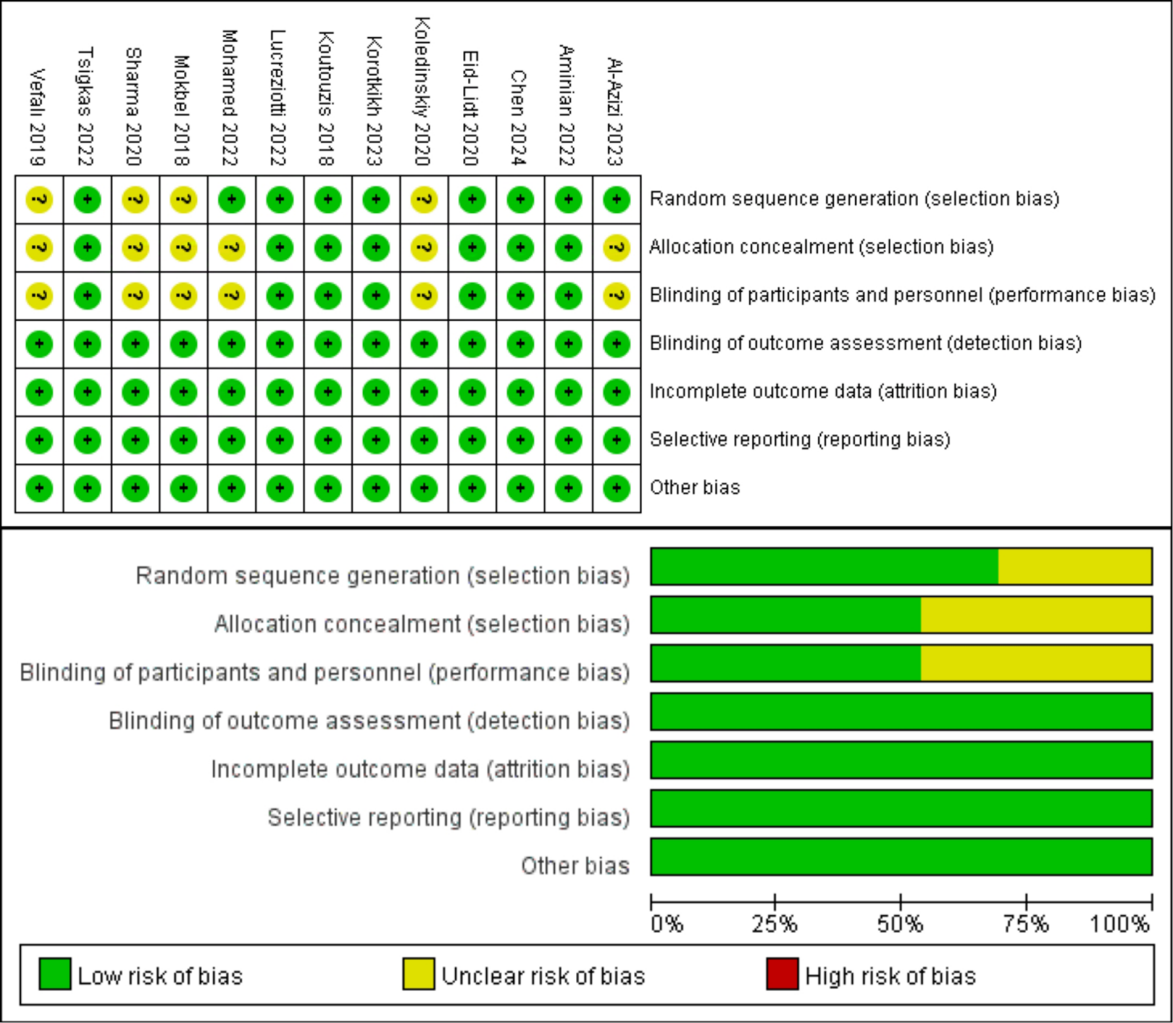
Risk of bias assessment for the included RCTs.

The NOS was used to evaluate the quality of the included prospective studies and retrospective studies. Among the 18 studies, 5 received a grade of 9, 8 received a rating of 8, and 5 received a rating of 7, indicating that all included studies were of good quality. Table 2 presents the specifics of the quality assessment by the NOS. The Cochrane Risk of Bias tool was used to assess the quality of 13 RCTs. Seven trials utilized blinded individuals or treatments, 13 trials conducted blindfold outcome assessments, 13 trials provided comprehensive results, 13 trials did not selectively disclose data, and 13 trials exhibited no risk of additional biases. Figure 2 presents comprehensive information on the quality assessment of RCTs.

**Table 2:** Quality assessment of the included studies by NOS.

Study	Selection	Comparability	Outcome	Total score
Bhambhani et al.(19)	★★★★	★★	★	7
Soydan et al.(23)	★★★★	★★	★★★	9
Roghani-Dehkordi et al. (24)	★★★★	★★	★★	8
Coughlan et al. (27)	★★★★	★★	★★	8
Li et al. (29)	★★★★	★★	★★	8
Lin et al. (30)	★★★★	★★	★★	8
Lu et al. (31)	★★★★	★★	★	7
Kis and Soydan (32)	★★★★	★★	★★★	9
Amin et al. (43)	★★★★	★★	★★	8
Hammami et al. (44)	★★★★	★★	★	7
Gajurel et al. (45)	★★★★	★★	★★	8
Aoi et al. (41)	★★★★	★	★★	7
Wang et al. (34)	★★★★	★★	★★★	9
Xu et al. (35)	★★★★	★★	★★	8
Chugh et al. (42)	★★★★	★★	★★★	9
Kaledin et al. (39)	★★★	★	★★★	7
Feng et al. (36)	★★★★	★★	★★★	9
Lalani et al. (37)	★★★★	★★	★★	8

### 3.4 Clinical outcomes

Table 3 summarizes the results of the meta-analysis for all clinical outcomes.

**Table 3:** Results of the meta-analysis.

Outcomes	No. of studies	Sample size		Heterogeneity		Overall effect size	95% CI of overall effect	*P*-value
		dTRA	TRA	*I*^2^ (%)	*P*-value			
RAO	22	6,132	6,199	16	0.25	OR = 0.41	0.34,0.50	<0.00001
Procedure success	9	1,735	1,739	32	0.17	OR = 0.41	0.30,0.56	<0.00001
Success rate of catheter puncture	17	6,885	7,268	87	<0.00001	OR = 0.44	0.27,0.71	0.0009
Success rate of a single attempt	6	1,340	1,345	93	<0.00001	OR = 0.46	0.20,1.08	0.07
Puncture point bleeding	8	2,580	2,614	88	<0.00001	OR = 0.48	0.19,1.20	0.12
Hematoma	19	5,435	5,393	42	0.03	OR = 0.67	0.56,0.80	<0.0001
Radical artery spasms	16	3,619	3,783	71	<0.00001	OR = 0.80	0.47,1.37	0.43
Puncture time	21	4,218	4,441	100	<0.00001	WMD = 0.72	0.58,0.86	<0.00001
Procedural time	13	3,178	3,198	98	<0.00001	WMD = −0.08	−2.18,2.01	0.94
Dosage of contrast medium	11	2,401	2,402	29	0.17	WMD = 0.28	−0.54,1.10	0.50
Hemostasis time	13	2,925	2,952	100	<0.00001	WMD = −0.43	−0.65,−0.20	0.0002

RAO, radial artery occlusion.

#### 3.4.1 RAO

**Figure 3:**
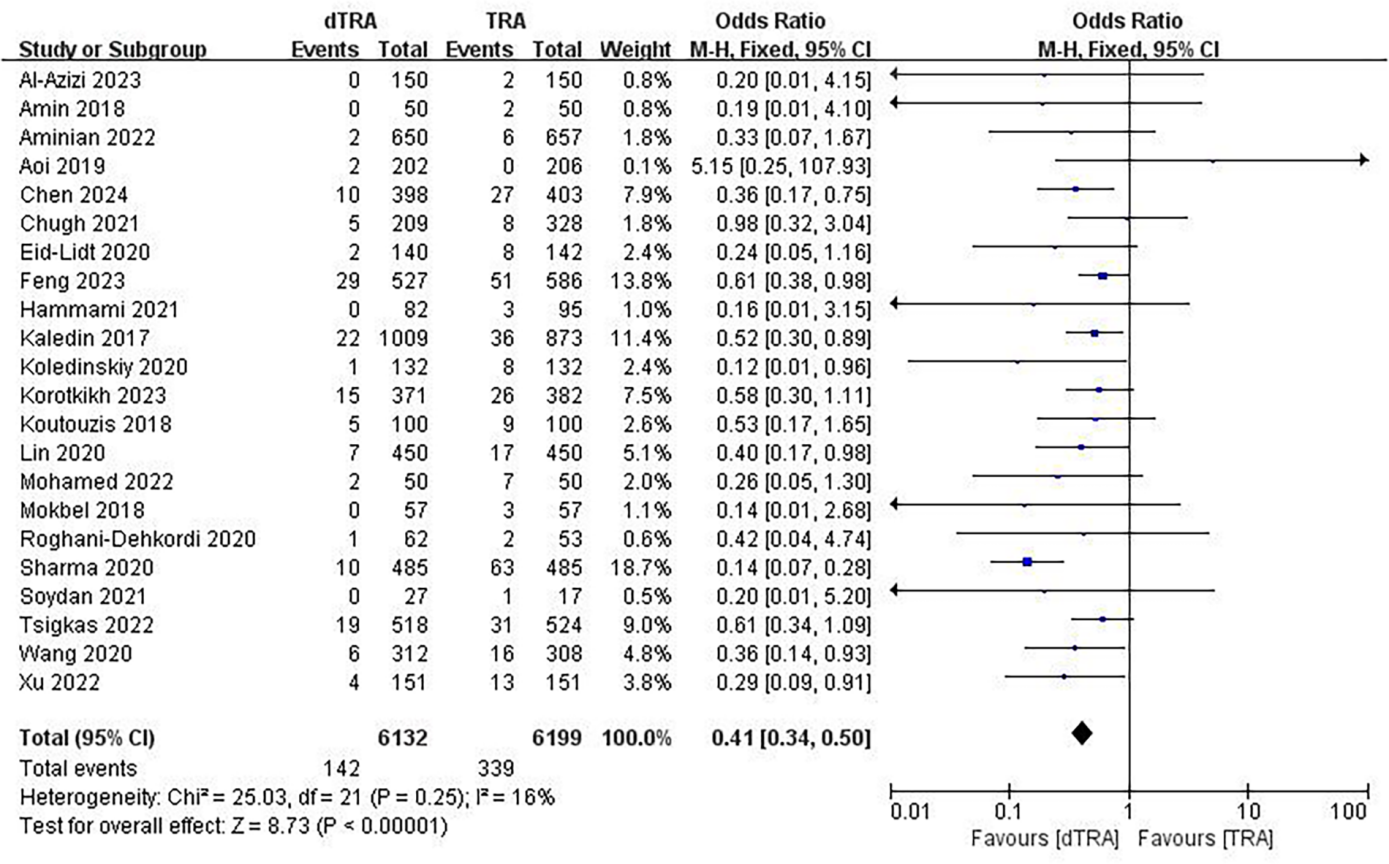
Forest plot of the meta-analysis for RAO.

A total of 22 studies (20–26, 28, 30, 33, 34, 41, 43, 44) documented RAO. The aggregated findings indicated that dTRA reduced the occurrence of RAO compared to cTRA (OR = 0.41, 95% CI: 0.34, 0.50, *P* < 0.00001, *I*^2^ = 16%) (Figure 3). A subgroup analysis of RAO in terms of time evaluation was performed. The subgroup analysis indicated that dTRA markedly reduced the occurrence of RAO during hospitalization (OR = 0.36, 95% CI: 0.25, 0.52, *P* < 0.00001, *I*^2^ = 0%) (Supplementary Figure S1) or RAO after 30 days (OR = 0.52, 95% CI: 0.37, 0.72, *P* < 0.00001, *I*^2^ = 15%) (Supplementary Figure S2).

#### 3.4.2 Success rate of catheter puncture

A total of 17 studies (19, 22, 26, 29–31, 34–37, 39–41, 45, 46, 48, 49) reported on the success rate of catheter puncture. The results showed a significant difference between the two groups, with dTRA exhibiting a lower success rate of catheter puncture compared to cTRA (OR = 0.44, 95% CI: 0.27, 0.71, *P* = 0.0009, *I*^2^ = 87%) (Supplementary Figure S3).

#### 3.4.3 Success rate of a single attempt

Six studies (21, 22, 25, 33, 35, 37) documented the success rate of a single attempt. No statistically significant difference was noted between the two groups in terms of the success rate of a single attempt (OR = 0.46, 95% CI: 0.20, 1.08, *P* = 0.07, *I*^2^ = 93%) (Supplementary Figure S4).

#### 3.4.4 Puncture point bleeding

Eight studies (20, 22, 28–30, 32, 41, 46) reported on puncture point bleeding. There was no statistically significant difference between the two groups in terms of puncture point bleeding (OR = 0.48, 95% CI: 0.19, 1.20, *P* = 0.12, *I*^2^ = 88%) (Supplementary Figure S5).

#### 3.4.5 Procedure success

Nine studies (19, 21, 22, 24, 29, 35, 44, 47, 49) reported on procedure success. The aggregate findings indicated that dTRA exhibited a markedly reduced procedural success compared to cTRA (OR = 0.41, 95% CI: 0.30, 0.56, *P* < 0.00001, I^2^ = 32%) (Supplementary Figure S6).

#### 3.4.6 Hematoma

A total of 19 studies (20–22, 25, 26, 28–30, 32, 33, 34, 36, 38, 39, 41, 43, 45, 48, 49) reported on the incidence of hematoma. The difference between dTRA and cTRA was statistically significant, with dTRA exhibiting a notably reduced incidence of hematoma compared to cTRA (OR = 0.67, 95% CI: 0.56, 0.80, *P* < 0.0001, *I*^2^ = 42%) (Supplementary Figure S7).

#### 3.4.7 Radial artery spasm

A total of 16 studies (20, 21, 25, 26, 32–35, 40–42, 44–46) reported on radial artery spasms. There was no statistically significant difference between the two groups in terms of radial artery spasms (OR = 0.80, 95% CI: 0.47, 1.37, *P* = 0.43, *I*^2^ = 71%) (Supplementary Figure S8).

#### 3.4.8 Puncture time (min)

A total of 21 studies (19, 20, 22, 24–26, 29, 30, 33, 35–38, 40–45, 48, 49) reported on puncture time. The pooled results revealed that dTRA had a significantly longer puncture time than cTRA (MD = 0.72, 95% CI: 0.58, 0.86, *P* < 0.00001, *I*^2^ = 100%) (Supplementary Figure S9).

#### 3.4.9 Procedural time (min)

A total of 13 studies (19, 20, 22, 24, 26, 27, 29, 33, 34, 35, 46, 48, 49) reported on procedural time. The two groups did not show a statistically significant difference in procedural time (MD = −0.08, 95% CI: −2.18, 2.01, *P* = 0.94, *I*^2^ = 98%) (Supplementary Figure S10).

#### 3.4.10 Dosage of contrast medium (ml)

A total of 11 studies (22, 24, 26, 27, 29, 33, 35, 40, 46, 48, 49) reported on the dosage of contrast medium. The two groups did not show a statistically significant difference in contrast medium dosage (MD = 0.28, 95% CI: −0.54, 1.10, *P* = 0.50, *I*^2^ = 29%) (Supplementary Figure S11).

#### 3.4.11 Hemostasis time (h)

A total of 13 studies (26–30, 33, 35, 38, 40, 41, 46, 48, 49) reported on hemostasis time. The pooled results showed that dTRA exhibited a significantly shorter hemostasis time than cTRA (MD = −0.43, 95% CI: −0.65,−0.20, *P* = 0.0002, *I*^2^ = 100%) (Supplementary Figure S12).

### 3.5 Subgroup analysis regarding patients with ACS

A subgroup analysis focusing on patients with ACS was performed (Table 4, Supplementary Figure S13–S21). The results showed that compared with cTRA, dTRA significantly reduced the incidence of RAO, puncture point bleeding, hematoma, and radical artery spasms but had significantly longer puncture time. There was no significant difference in the success rate of catheter puncture, procedural time, dosage of contrast medium, and hemostasis time.

**Table 4:** Results of the subgroup meta-analysis involving patients with ACS.

Outcomes	No. of studies	Sample size		Heterogeneity		Overall effect size	95% CI of overall effect	*P*-value
		dTRA	TRA	*I*^2^ (%)	*P*-value			
RAO	5	1,653	1,513	0	0.64	OR = 0.41	0.26,0.63	<0.0001
Success rate of catheter puncture	6	3,405	3,728	86	<0.00001	OR = 0.46	0.18,1.22	0.12
Puncture point bleeding	2	273	290	0	0.76	OR = 0.31	0.10,0.89	0.03
Hematoma	7	1,876	1,757	0	0.60	OR = 0.36	0.19,0.67	0.001
Radical artery spasms	4	612	607	0	0.47	OR = 0.50	0.28,0.89	0.02
Puncture time	6	540	545	100	<0.00001	WMD = 1.16	0.57,1.74	<0.0001
Procedural time	3	462	462	77	0.01	WMD = 0.21	−2.19,2.61	0.86
Dosage of contrast medium	3	252	257	0	0.87	WMD = 0.11	−5.93,6.16	0.97
Hemostasis time	6	624	631	100	<0.00001	WMD = 0.26	−0.04,0.56	0.09

RAO, radial artery occlusion.

### 3.6 Subgroup analysis regarding only RCTs

A RCT subgroup analysis was performed (Table 5, Supplementary Figure S22–S32). Compared with cTRA, dTRA significantly reduced the incidence of RAO, hematoma, and hemostasis. However, dTRA had a significantly lower procedure success rate, a lower success rate of catheter puncture, and a longer puncture time. There was no significant difference between the two approaches in terms of success rate of a single attempt, radial artery spasm, hemostasis time, puncture site bleeding, procedural time, or dosage of contrast medium.

**Table 5:** Results of the subgroup meta-analysis involving only RCTs.

Outcomes	No. of studies	Sample size		Heterogeneity		Overall effect size	95% CI of overall effect	*P*-value
		dTRA	TRA	*I*^2^ (%)	*P*-value			
RAO	11	3,051	3,082	34	0.13	OR = 0.34	0.25,0.45	<0.00001
Procedure success	4	990	995	46	0.14	OR = 0.48	0.30,0.76	0.002
Success rate of catheter puncture	6	1,818	1,840	90	<0.00001	OR = 0.34	0.14,0.82	0.02
Success rate of a single attempt	4	1,123	1,130	96	<0.00001	OR = 0.56	0.18,1.74	0.32
Puncture point bleeding	4	1,569	1,592	92	<0.00001	OR = 0.33	0.08,1.34	0.12
Hematoma	10	2,444	2,472	50	0.03	OR = 0.72	0.58,0.89	0.002
Radical artery spasms	8	2,498	2,525	84	<0.00001	OR = 0.59	0.25,1.35	0.21
Puncture time	9	1,895	1,934	100	<0.00001	WMD = 0.65	0.44,0.85	<0.00001
Procedural time	7	2,164	2,197	99	<0.00001	WMD = 0.39	−2.68,3.45	0.80
Dosage of contrast medium	7	1,799	1,809	0	0.68	WMD = 0.49	−0.34,1.32	0.25
Hemostasis time	8	1,733	1,756	100	<0.00001	WMD = 0.23	−0.05,0.51	0.11

RAO, radial artery occlusion.

### 3.7 Publication bias

**Figure 4:**
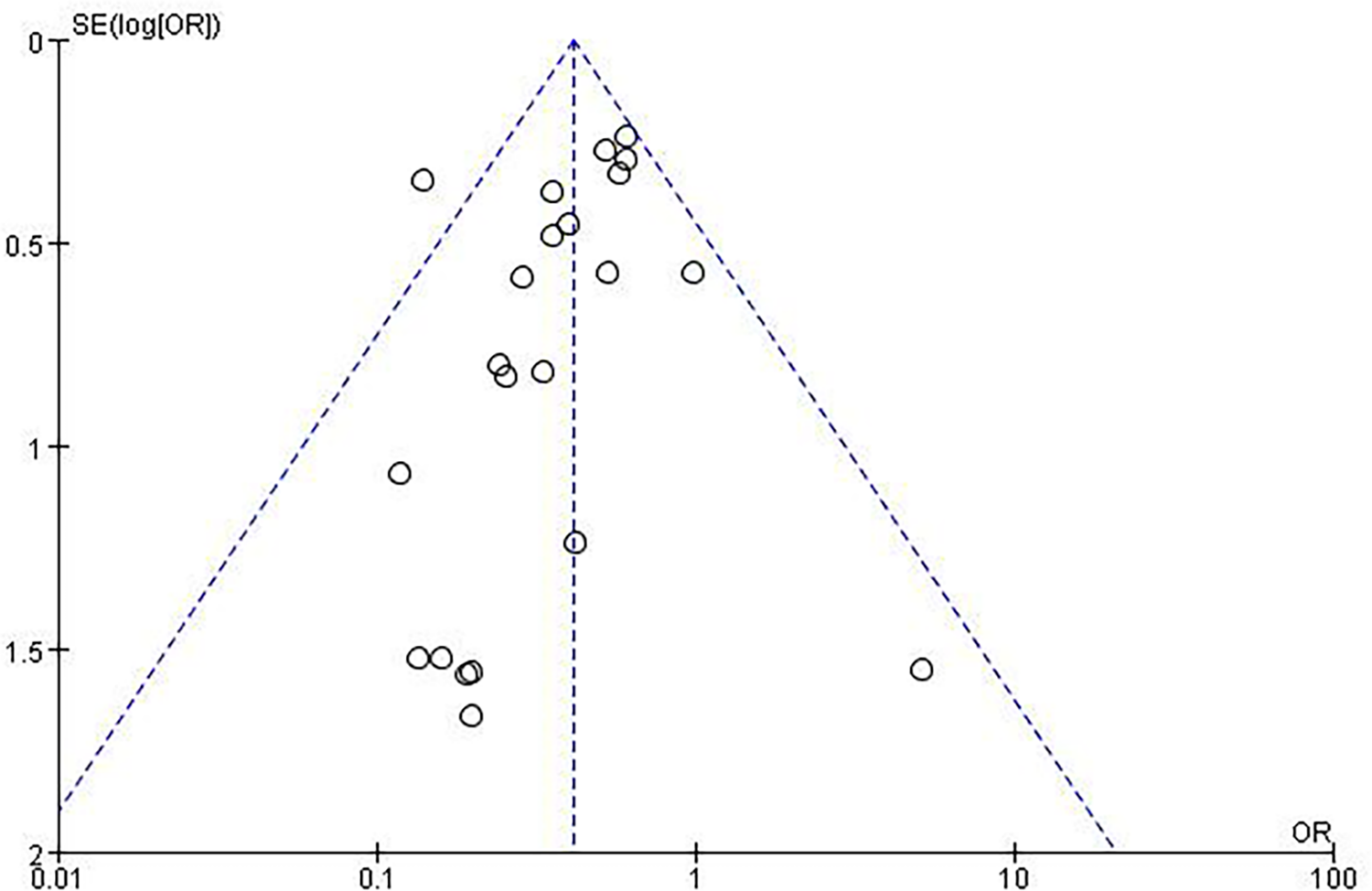
Funnel plot for RAO.

An evaluation of publication bias related to RAO was conducted using a funnel plot (Figure 4). The bilaterally symmetric funnel plot of the RAO did not reveal any significant evidence of publication bias.

## 4 Discussion

As TRA has a lower risk of consequences than transfemoral access, including death in patients presenting with acute coronary syndromes, it is presently the preferred access method for coronary operations (50, 51). However, TRA is not a panacea—RAO remains the “Achilles’ heel” of this method. In addition, because the hand has two sets of blood vessels coming from the palmar arch, RAO rarely shows up in the real world. However, RAO prevents the radial artery form being used for future invasive treatments or coronary artery bypass grafting. Estimates of how often it happens remain uncertain because the rates depend a lot on patient characteristics, procedural factors, anticoagulation protocols, and hemostasis techniques (52). DTRA through the anatomic snuffbox has emerged as a new method for CAG and PCI in the last few years (10). Meanwhile, several investigations have shown that recanalizing radial artery stenosis or occlusion via dTRA is both safe and practical (53–55). Moreover, no studies have reported that dTRA is associated with a higher radiation dose (56). The reduced size of the distal radial artery heightens the difficulty of puncture (55). Therefore, we conducted this meta-analysis to compare the efficacy and safety of dTRA vs. cTRA for coronary diagnostic or interventional catheterization.

Our results indicated that dTRA substantially decreases the incidence of RAO compared to cTRA. Thrombosis is a significant contributor to the development of RAO, with its pathogenesis and etiology involving abnormalities in blood flow (stasis), vascular endothelial damage, and hypercoagulability (57). The smaller diameter of the radial artery, the lower amount of nitric oxide released during puncture, endothelial damage, and reduced blood flow due to sheaths and catheters, along with the increase in intimal hyperplasia and intima-media thickness due to constant intubation, contribute to the higher likelihood of RAO with cTRA (58, 59). Among these factors, endothelial damage is the most significant cause of RAO. The repeated introduction and withdrawal of catheters can easily damage the arterial intima, leading to RAO (60). While most RAO cases are asymptomatic, the condition makes it harder to use for catheterizations and as a conduit in people who need coronary artery bypass grafting or radial arteriovenous fistula formation in people with kidney dysfunction. As a result, RAO prevention and management are critical. Preinterventional visualization of the radial artery in both limbs with Doppler ultrasonography is recommended to evaluate the characteristics of the radial artery. It is particularly beneficial for selecting an artery with a larger diameter and determining the size and depth of the artery. Consequently, arterial cannulation is facilitated, diminishing the need for multiple puncture attempts. This imaging modality can assist in determining the suitable sheath size to decrease the sheath-to-artery mismatch, reducing the incidence of RAO (61). In addition, anticoagulants can also effectively prevent and treat thrombosis, thus averting the beginning of RAO (62), including novel oral anticoagulants (63). Furthermore, the lack of blood flow during hemostasis dramatically also increases the probability of RAO, as flow interruption at this stage is a key predictor of such occlusion (64). Studies have shown that interventions like patent hemostasis (65), nitroglycerin administration through the sheath before its removal (66), and ipsilateral ulnar artery compression during radial artery hemostasis (67) can reduce the incidence of postcatheterization RAO (68). In this context, dTRA may help preserve the potency of forearm radial artery during hemostatic compression or obstruction at the puncture site. Maintaining blood flow in the distal radial access is crucial to preventing proximal thrombus formation and keeping the forearm radial artery open after TRA (21). This is made easier by the collateralization of blood vessels. In the context of forearm radial artery obstruction, dTRA is preferable to cTRA.

Compared to cTRA, dTRA decreases the incidence of hematoma, as shown in this current meta-analysis. Hematoma formation is likely caused by poor compression positioning, dual antiplatelet therapy, heparin use, advanced age, delicate skin, and multiple puncture attempts. A standardized technique for compression duration and a specialized compression device may help reduce the incidence of possible bleeding issues (69, 70). Our findings indicate that dTRA can reduce hemostasis time compared to cTRA; this may suggest a decreased occurrence of hematoma in dTRA, attributable to the anatomical structure of the distal radial artery, which has a smaller diameter and is situated over a bony basis formed by the scaphoid and trapezium carpal bones (26).

Our results indicated that dTRA has a lower procedural success rate and effective catheter puncture rate compared to cTRA, which necessitates a longer puncture time. Multiple variables may contribute to their occurrence: (1) the radial artery in the AS is narrower than that at the wrist (71), complicating puncture or sheath insertion following a successful puncture; (2) the distal radial artery frequently displays tortuosity, which can readily result in the unsuccessful insertion of the guidewire and sheath into the radial artery; and (3) dTRA is a novel approach for CAG and PCI, and many operators lack expertise in puncture management and must surmount the learning curve (29). The standard ultrasound-guided approach and enhanced proficiency in the puncture technique may elevate the success rate over time and reduce the risk of puncture-induced vasospasm (44). Lee et al. (69) discovered that the puncture duration progressively stabilized after roughly 150 distal radial artery punctures. A multicenter study indicated that after treating 150 patients, the learning curve achieved an optimal average of two puncture attempts, each lasting under 30 s (72). Deora et al. indicated that, after a specific learning curve (minimum of 50 successful punctures), operational time could be further reduced with further expertise, particularly in assessing the arterial entry via the distal radial artery (73). Therefore, the learning curve varies among different operators, depending on their skills in radial artery access puncture. Mori et al. found that ultrasound-guided dTRA for CAG or PCI exhibited a reduced failure rate compared to traditional dTRA (74). Two meta-analyses (75, 76) compared ultrasound-guided puncture with a palpation-based blind puncture for traditional radial artery access, indicating that ultrasound-guided puncture yields a greater first-pass success rate, reduced puncture time, and decreased hematoma development. Furthermore, although the success rate of catheter puncture for dTRA is lower than that for cTRA (2.89% lower), the success rate remains notably high (91.93% vs. 94.82%). Based on the lower complication rate of dTRA, predominantly with a lower incidence of RAO, we preferentially recommend dTRA for interventional surgeries in coronary heart disease. The cTRA procedure may serve as an alternative in cases where dTRA is unsuccessful.

A subgroup analysis focusing on patients with ACS was performed in our study. Compared with cTRA, dTRA significantly reduced the incidence of RAO, puncture site bleeding, hematoma, and radical artery spasms but had a significantly longer puncture time (WMD = 1.16 min). Notably, there was no significant difference between the two groups in the total procedural time. In the acute environment, every minute is critical, particularly for patients with ST-segment elevation myocardial infarction, and dTRA necessitates additional time for effective arterial access. Apostolos et al. have discussed the use of dTRA in patients with acute coronary syndrome (77). dTRA has been linked to expedited hemostasis, quicker patient mobilization, and a reduction in local sequelae, including substantial hematomas or compartment syndrome. Considering these factors, the prior delays from dTRA should not impede its initial application in patients with acute coronary syndrome. In addition, Desora et al. indicated that, after a specific learning curve, operational time can be further minimized with further expertise, particularly in assessing the arterial entry via the distal radial artery (73).

Although previous meta-analyses (50, 78–80) have compared dTRA and cTRA, the number of articles included in these meta-analyses was relatively low due to insufficient search strategies or shorter study durations. To our knowledge, this updated meta-analysis includes the largest number of studies comparing the outcomes of dTRA vs. cTRA for cardiovascular interventional diagnosis and/or treatment. To manage confounding variables, we performed subgroup analyses focusing on patients with ACS or RCTs. This may result in a more trustworthy judgment. The findings of our meta-analysis provided valuable perspectives on the clinical selection of interventional surgical techniques, which enhance clinical practice and research in the area of cardiovascular interventional diagnosis and/or treatment. However, we acknowledge the possible shortcomings of our study. First, a large number of the studies were not randomized controlled trials, which resulted in a relatively poor quality of evidence and credibility. Second, we could not manage confounding variables, including varying inclusion criteria, population disparities, and the level of experience of surgeons, which may lead to heterogeneity among the trials and introduce bias. Consequently, more clinical outcomes reported by prospective randomized controlled studies are essential to further validate the benefits of dTRA.

In conclusion, our results demonstrated that dTRA is a feasible and safe approach for coronary angiography and percutaneous coronary intervention. Compared with cTRA, dTRA significantly reduces postoperative complications, particularly the occurrence of RAO, and expedites the period to hemostasis.

## Data Availability

The datasets presented in this study can be found in online repositories. The names of the repository/repositories and accession number(s) can be found in the article/Supplementary Material.
