# Supplementary material for: Efficacy and safety of distal transradial access for coronary angiography and percutaneous coronary intervention: a meta-analysis
Source: Front Cardiovasc Med. 2025 Mar 18;12:1530995. doi: 10.3389/fcvm.2025.1530995 (PMC11959052; doi:10.3389/fcvm.2025.1530995)
Supplement: Supplementary file 1 [file Datasheet1.pdf]

Supplementary Tables

Supplementary Table 1. The details of the searching record in Pubmed

1	((((((((Coronary Disease[Title/Abstract]) OR (Coronary Diseases[Title/Abstract])) OR (Disease, Coronary[Title/Abstract])) OR (Diseases, Coronary[Title/Abstract])) OR (Coronary Heart Disease[Title/Abstract])) OR (Coronary Heart Diseases[Title/Abstract])) OR (Disease, Coronary Heart[Title/Abstract])) OR (Diseases, Coronary Heart[Title/Abstract])) OR (Heart Disease, Coronary[Title/Abstract])) OR (Heart Diseases, Coronary[Title/Abstract])	79883
2	(((Coronary angiography[Title/Abstract]) OR (Angiography, Coronary[Title/Abstract])) OR (Angiographies, Coronary[Title/Abstract])) OR (Coronary Angiographies[Title/Abstract])	39934
3	((((((((((Percutaneous Coronary Intervention[Title/Abstract]) OR (Coronary Intervention, Percutaneous[Title/Abstract])) OR (Coronary Interventions, Percutaneous[Title/Abstract])) OR (Intervention, Percutaneous Coronary[Title/Abstract])) OR (Interventions, Percutaneous Coronary[Title/Abstract])) OR (Percutaneous Coronary Interventions[Title/Abstract])) OR (Percutaneous Coronary Revascularization[Title/Abstract])) OR (Coronary Revascularization, Percutaneous[Title/Abstract])) OR (Coronary Revascularizations, Percutaneous[Title/Abstract])) OR (Percutaneous Coronary Revascularizations[Title/Abstract])) OR (Revascularization, Percutaneous Coronary[Title/Abstract])) OR (Revascularizations, Percutaneous Coronary[Title/Abstract])	49884
4	((((Radial Artery[Title/Abstract]) OR (Arteries, Radial[Title/Abstract])) OR (Artery, Radial[Title/Abstract])) OR (Radial Arteries[Title/Abstract])) OR (Transradial[Title/Abstract])	12232
5	(Snuff box[Title/Abstract]) OR (Distal radial artery[Title/Abstract])	401
6	#1 AND (#2 OR #3) AND #4 AND #5	2

Supplementary Table 2. The details of the searching record in Web of science

1	((((((((TS=(Coronary Disease)) OR TS=(Coronary Diseases)) OR TS=(Disease, Coronary)) OR TS=(Diseases, Coronary)) OR TS=(Coronary Heart Disease)) OR TS=(Coronary Heart Diseases)) OR TS=(Disease, Coronary Heart)) OR TS=(Diseases, Coronary Heart)) OR TS=(Heart Disease, Coronary)) OR TS=(Heart Diseases, Coronary)	832,386
2	(((TS=(Coronary angiography)) OR TS=(Angiography, Coronary)) OR TS=(Angiographies, Coronary)) OR TS=(Coronary Angiographies)	165277
3	((((((((((TS=(Percutaneous Coronary Intervention)) OR TS=(Coronary Intervention, Percutaneous)) OR TS=(Coronary Interventions, Percutaneous)) OR TS=(Intervention, Percutaneous Coronary)) OR TS=(Interventions,	115930

	Percutaneous Coronary)) OR TS=(Percutaneous Coronary Interventions)) OR TS=(Percutaneous Coronary Revascularization)) OR TS=(Coronary Revascularization, Percutaneous)) OR TS=(Coronary Revascularizations, Percutaneous)) OR TS=(Percutaneous Coronary Revascularizations)) OR TS=(Revascularization, Percutaneous Coronary)) OR TS=(Revascularizations, Percutaneous Coronary)	
4	((((TS=(Radial Artery)) OR TS=(Arteries, Radial)) OR TS=(Artery, Radial)) OR TS=(Radial Arteries)) OR TS=(Transradial)	35623
5	(TS=(Snuff box)) OR TS=(Distal radial artery)	4176
6	#1 AND (#2 OR #3) AND #4 AND #5	427

Supplementary Table 3. The details of the searching record in Embase

1	'Coronary Disease':ab,ti,kw OR 'Coronary Diseases':ab,ti,kw OR 'Disease, Coronary':ab,ti,kw OR 'Diseases, Coronary':ab,ti,kw OR 'Coronary Heart Disease':ab,ti,kw OR 'Coronary Heart Diseases':ab,ti,kw OR 'Coronary Heart Diseases':ab,ti,kw OR 'Disease, Coronary Heart':ab,ti,kw OR 'Diseases, Coronary Heart':ab,ti,kw OR 'Heart Disease, Coronary':ab,ti,kw OR 'Heart Diseases, Coronary':ab,ti,kw	112929
2	'Coronary angiography':ab,ti,kw OR 'Angiography, Coronary':ab,ti,kw OR 'Angiographies, Coronary':ab,ti,kw OR 'Coronary Angiographies':ab,ti,kw	69911
3	'Percutaneous Coronary Intervention':ab,ti,kw OR 'Coronary Intervention, Percutaneous':ab,ti,kw OR 'Coronary Interventions, Percutaneous':ab,ti,kw OR 'Intervention, Percutaneous Coronary':ab,ti,kw OR 'Interventions, Percutaneous Coronary':ab,ti,kw OR 'Percutaneous Coronary Interventions':ab,ti,kw OR 'Percutaneous Coronary Revascularization':ab,ti,kw OR 'Coronary Revascularization, Percutaneous':ab,ti,kw OR 'Coronary Revascularizations, Percutaneous':ab,ti,kw OR 'Percutaneous Coronary Revascularizations':ab,ti,kw OR 'Revascularization, Percutaneous Coronary':ab,ti,kw OR 'Revascularizations, Percutaneous Coronary ':ab,ti,kw	83232
4	'Radial Artery':ab,ti,kw OR 'Arteries, Radial':ab,ti,kw OR 'Artery, Radial':ab,ti,kw OR 'Radial Arteries':ab,ti,kw OR 'Transradial':ab,ti,kw	18825
5	'Snuff box':ab,ti,kw OR 'Distal radial artery':ab,ti,kw	609
6	#1 AND (#2 OR #3) AND #4 AND #5	3

Supplementary Table 4. The details of the searching record in cochrane library

1	(Coronary Disease OR Coronary Diseases OR Disease, Coronary OR Diseases, Coronary OR Coronary Heart Disease OR Coronary Heart Diseases OR Disease,	44381

	Coronary Heart OR Diseases, Coronary Heart OR Heart Disease, Coronary OR Heart Diseases, Coronary):ab,ti,kw	
2	(Coronary angiography OR Angiography, Coronary OR Angiographies, Coronary OR Coronary Angiographies):ab,ti,kw	12066
3	(Percutaneous Coronary Intervention OR Coronary Intervention, Percutaneous OR Coronary Interventions, Percutaneous OR Intervention, Percutaneous Coronary OR Interventions, Percutaneous Coronary OR Percutaneous Coronary Interventions OR Percutaneous Coronary Revascularization OR Coronary Revascularization, Percutaneous OR Coronary Revascularizations, Percutaneous OR Percutaneous Coronary Revascularizations OR Revascularization, Percutaneous Coronary OR Revascularizations, Percutaneous Coronary):ab,ti,kw	14377
4	(Radial Artery OR Arteries, Radial OR Artery, Radial OR Radial Arteries OR Transradial):ab,ti,kw	3346
5	(Snuff box OR Distal radial artery):ab,ti,kw	290
6	#1 AND (#2 OR #3) AND #4 AND #5	55
