# Supplementary material for: Efficacy and safety of distal transradial access for coronary angiography and percutaneous coronary intervention: a meta-analysis
Source: Front Cardiovasc Med. 2025 Mar 18;12:1530995. doi: 10.3389/fcvm.2025.1530995 (PMC11959052; doi:10.3389/fcvm.2025.1530995)

Supplementary Figures

Figure S1. Forest plot of the meta-analysis for RAO during

Study or Subgroup	dTRA Events	dTRA Total	cTRA Events	cTRA Total	Weight	Odds Ratio M-H, Fixed, 95% CI
Amin2018	0	50	2	50	2.4%	0.19 [0.01, 4.10]
Aminian2022	2	650	6	657	5.7%	0.33 [0.07, 1.67]
Aoi2019	2	202	0	206	0.5%	5.15 [0.25, 107.93]
Chen2024	10	398	27	403	24.9%	0.36 [0.17, 0.75]
Chugh2021	5	209	8	328	5.8%	0.98 [0.32, 3.04]
Eid-Lidt2020	1	140	12	142	11.2%	0.08 [0.01, 0.61]
Hammami2021	0	82	3	95	3.1%	0.16 [0.01, 3.15]
Koutouzis2018	5	100	9	100	8.1%	0.53 [0.17, 1.65]
Mohamed2022	2	50	7	50	6.4%	0.26 [0.05, 1.30]
Mokbel2018	0	57	3	57	3.3%	0.14 [0.01, 2.68]
Soydan2021	0	27	1	17	1.7%	0.20 [0.01, 5.20]
Wang2020	6	312	16	308	15.0%	0.36 [0.14, 0.93]
Xu2022	4	151	13	151	12.0%	0.29 [0.09, 0.91]
**Total (95% CI)**		**2428**		**2564**	**100.0%**	**0.36 [0.25, 0.52]**
Total events	37		107			

Heterogeneity: Chi² = 9.82, df = 12 (P = 0.63); I² = 0%
Test for overall effect: Z = 5.39 (P < 0.00001)

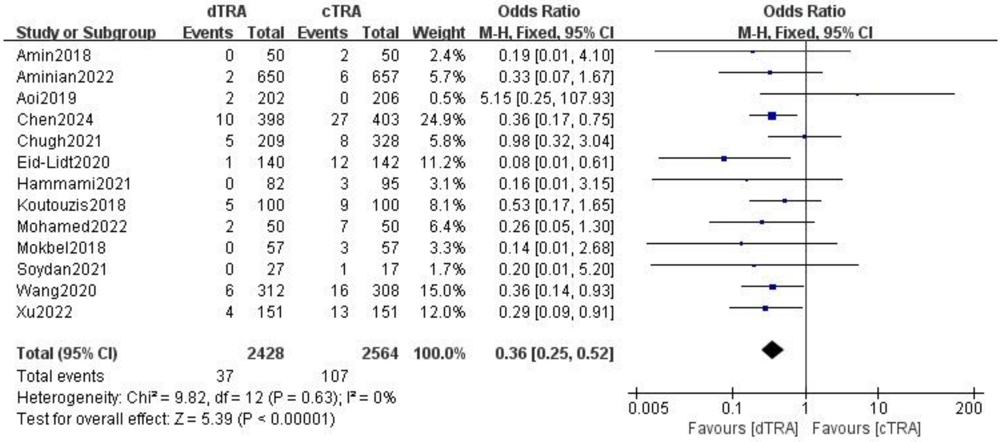

Figure S2. Forest plot of the meta-analysis for RAO after 30 days.

Study or Subgroup	dTRA Events	dTRA Total	cTRA Events	cTRA Total	Weight	Odds Ratio M-H, Fixed, 95% CI
Al-Azizi2023	0	150	2	150	2.4%	0.20 [0.01, 4.15]
Chen2024	3	361	12	365	11.3%	0.25 [0.07, 0.88]
Chugh2021	6	209	7	328	5.0%	1.36 [0.45, 4.09]
Eid-Lidt2020	1	140	8	142	7.5%	0.12 [0.01, 0.98]
Feng2023	29	527	51	586	43.4%	0.61 [0.38, 0.98]
Roghani-Dehkordi2020	1	62	2	53	2.0%	0.42 [0.04, 4.74]
Tsigkas2022	15	518	31	524	28.5%	0.47 [0.25, 0.89]
**Total (95% CI)**		**1967**		**2148**	**100.0%**	**0.52 [0.37, 0.72]**
Total events	55		113			

Heterogeneity: Chi² = 7.05, df = 6 (P = 0.32); I² = 15%
Test for overall effect: Z = 3.93 (P < 0.0001)

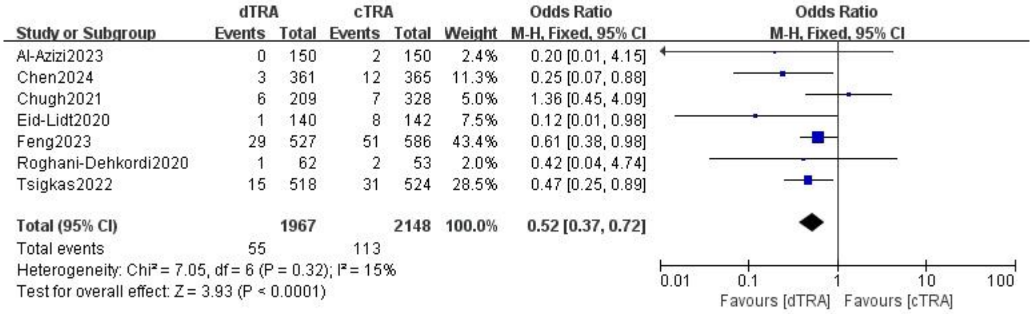

Figure S3. Forest plot of the meta-analysis for successful catheter puncture.

Study or Subgroup	dTRA Events	dTRA Total	TRA Events	TRA Total	Weight	Odds Ratio M-H, Random, 95% CI
Aminian 2022	525	650	528	656	8.2%	1.02 [0.77, 1.34]
Aoi 2019	201	202	204	206	2.7%	1.97 [0.18, 21.90]
Bhambhani 2020	77	100	93	100	6.5%	0.25 [0.10, 0.62]
Chen 2024	382	398	397	403	6.4%	0.36 [0.14, 0.93]
Feng 2023	455	527	566	586	7.7%	0.22 [0.13, 0.37]
Gajurel 2018	80	82	79	82	3.9%	1.52 [0.25, 9.34]
Kaledin 2017	2704	2775	2975	3099	8.2%	1.59 [1.18, 2.13]
Lalani 2024	61	66	63	64	3.1%	0.19 [0.02, 1.71]
Li 2022	320	342	330	342	7.1%	0.53 [0.26, 1.09]
Lin 2020	432	450	435	450	7.2%	0.83 [0.41, 1.66]
Lu 2020	34	40	40	40	2.1%	0.07 [0.00, 1.21]
Lucreziotti 2022	66	100	95	104	6.9%	0.18 [0.08, 0.41]
Mohamed 2022	37	50	48	50	4.5%	0.12 [0.03, 0.56]
Tsigkas 2022	408	518	497	524	7.9%	0.20 [0.13, 0.31]
Vefalı 2019	97	102	99	103	5.1%	0.78 [0.20, 3.01]
Wang 2020	296	312	295	308	7.0%	0.82 [0.39, 1.72]
Xu 2022	136	151	148	151	5.4%	0.18 [0.05, 0.65]
**Total (95% CI)**		**6865**		**7268**	**100.0%**	**0.44 [0.27, 0.71]**
Total events	6311		6892			

Heterogeneity: Tau² = 0.72; Chi² = 120.08, df = 16 (P < 0.00001); I² = 87%
Test for overall effect: Z = 3.33 (P = 0.0009)

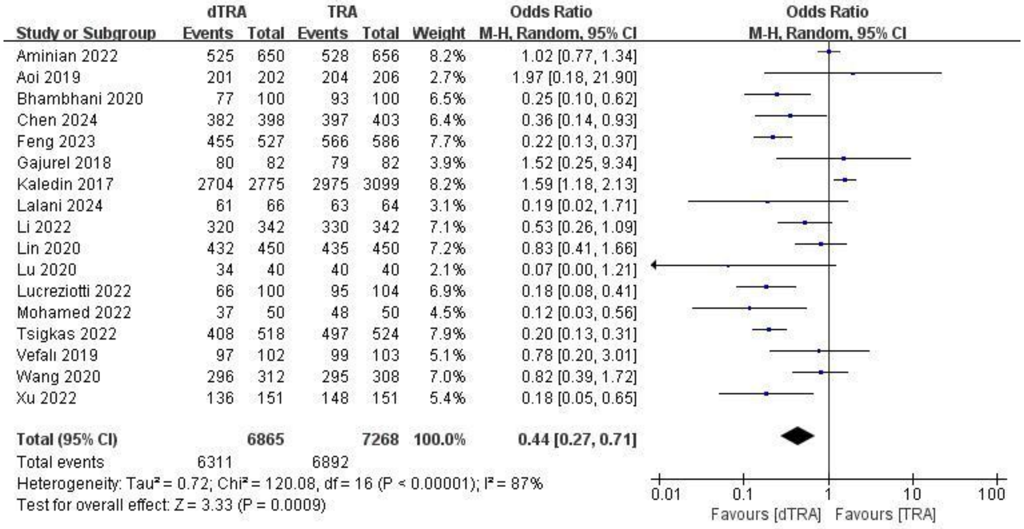

Figure S4. Forest plot of the meta-analysis for puncture in a single attempt.

Study or Subgroup	dTRA Events	dTRA Total	TRA Events	TRA Total	Weight	Odds Ratio M-H, Random, 95% CI
Chen 2024	282	398	338	403	17.6%	0.47 [0.33, 0.66]
Eid-Lidt 2020	67	140	35	142	17.0%	2.81 [1.69, 4.65]
Koutouzis 2018	27	100	41	100	16.6%	0.53 [0.29, 0.96]
Lalani 2024	46	66	54	64	15.3%	0.43 [0.18, 1.00]
Sharma 2020	378	485	466	485	17.0%	0.14 [0.09, 0.24]
Xu 2022	104	151	137	151	16.4%	0.23 [0.12, 0.43]
**Total (95% CI)**		**1340**		**1345**	**100.0%**	**0.46 [0.20, 1.08]**
Total events	904		1071			

Heterogeneity: Tau² = 1.01; Chi² = 74.06, df = 5 (P < 0.00001); I² = 93%
Test for overall effect: Z = 1.79 (P = 0.07)

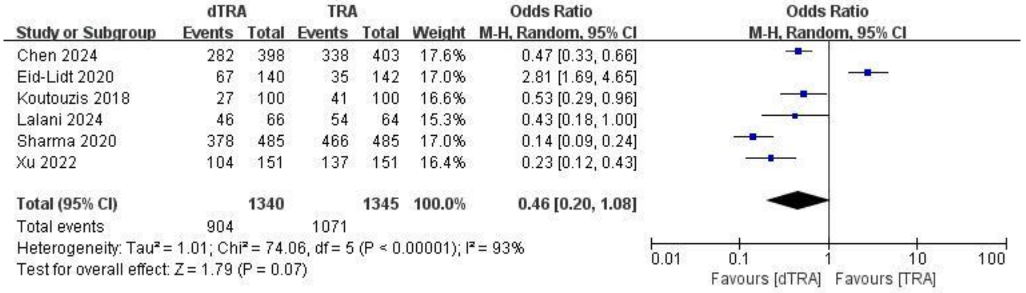

Figure S5. Forest plot of the meta-analysis for puncture point bleeding.

Study or Subgroup	dTRA Events	dTRA Total	TRA Events	TRA Total	Weight	Odds Ratio M-H, Random, 95% CI
Al-Azizi 2023	0	150	2	150	5.8%	0.20 [0.01, 4.15]
Aminian 2022	44	650	36	657	15.7%	1.25 [0.80, 1.97]
Aoi 2019	20	202	3	206	12.6%	7.44 [2.17, 25.44]
Chen 2024	6	398	24	403	14.0%	0.24 [0.10, 0.60]
Kis 2020	1	17	3	24	7.8%	0.44 [0.04, 4.61]
Korotkikh 2023	14	371	83	382	15.3%	0.14 [0.08, 0.25]
Li 2022	6	342	25	342	14.0%	0.23 [0.09, 0.56]
Lin 2020	11	450	29	450	14.8%	0.36 [0.18, 0.74]
**Total (95% CI)**		**2580**		**2614**	**100.0%**	**0.48 [0.19, 1.20]**
Total events	102		205			

Heterogeneity: Tau² = 1.32; Chi² = 58.60, df = 7 (P < 0.00001); I² = 88%
Test for overall effect: Z = 1.57 (P = 0.12)

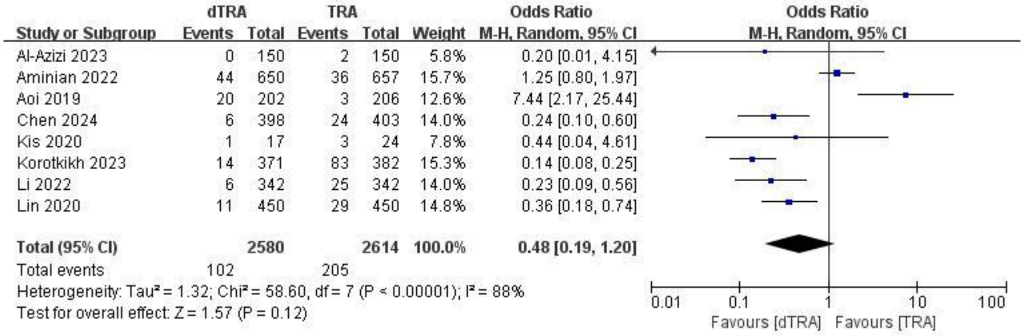

Figure S6. Forest plot of the meta-analysis for procedure success.

Study or Subgroup	dTRA Events	dTRA Total	TRA Events	TRA Total	Weight	Odds Ratio M-H, Fixed, 95% CI
Bhambhani 2020	77	100	93	100	16.9%	0.25 [0.10, 0.62]
Chen 2024	380	398	394	403	14.0%	0.48 [0.21, 1.09]
Hammami 2021	72	82	93	95	8.3%	0.15 [0.03, 0.73]
Li 2022	311	342	330	342	23.7%	0.36 [0.18, 0.72]
Mohamed 2022	37	50	48	50	9.9%	0.12 [0.03, 0.56]
Mokbel 2018	51	57	50	57	4.2%	1.19 [0.37, 3.79]
Roghani-Dehkordi 2020	62	70	53	56	5.3%	0.44 [0.11, 1.74]
Sharma 2020	466	485	475	485	14.7%	0.52 [0.24, 1.12]
Xu 2022	147	151	146	151	3.1%	1.26 [0.33, 4.78]
**Total (95% CI)**		**1735**		**1739**	**100.0%**	**0.41 [0.30, 0.56]**
Total events	1603		1682			

Heterogeneity: Chi² = 11.70, df = 8 (P = 0.17); I² = 32%
Test for overall effect: Z = 5.50 (P < 0.00001)

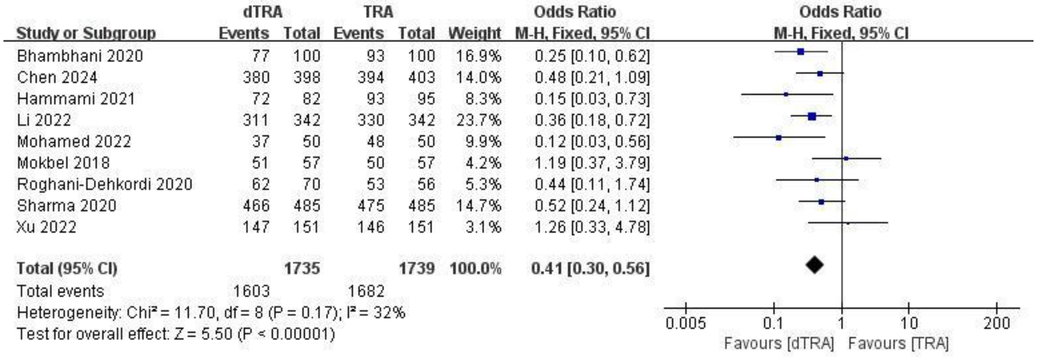

Figure S7. Forest plot of the meta-analysis for hematoma.

Study or Subgroup	dTRA Events	dTRA Total	TRA Events	TRA Total	Weight	Odds Ratio M-H, Fixed, 95% CI
Al-Azizi 2023	0	150	1	150	0.5%	0.33 [0.01, 8.19]
Amin 2018	0	50	2	50	0.9%	0.19 [0.01, 4.10]
Aoi 2019	7	202	5	206	1.7%	1.44 [0.45, 4.62]
Chen 2024	18	398	18	403	6.0%	1.01 [0.52, 1.98]
Eid-Lidt 2020	23	140	23	142	6.7%	1.02 [0.54, 1.91]
Feng 2023	18	527	48	586	15.4%	0.40 [0.23, 0.69]
Gajurel 2018	0	82	2	82	0.9%	0.20 [0.01, 4.13]
Kaledin 2017	2	1009	2	873	0.8%	0.86 [0.12, 6.15]
Kis 2020	0	17	2	24	0.7%	0.26 [0.01, 5.71]
Koledinskiy 2020	1	132	9	132	3.1%	0.10 [0.01, 0.84]
Korotkikh 2023	82	371	130	382	35.0%	0.55 [0.40, 0.76]
Koutouzis 2018	1	100	1	100	0.3%	1.00 [0.06, 16.21]
Li 2022	2	342	11	342	3.8%	0.18 [0.04, 0.80]
Lin 2020	11	450	8	450	2.7%	1.38 [0.55, 3.47]
Lucreziotti 2022	4	100	9	104	3.0%	0.44 [0.13, 1.48]
Mohamed 2022	0	50	5	50	1.9%	0.08 [0.00, 1.52]
Sharma 2020	48	485	38	485	12.0%	1.29 [0.83, 2.02]
Tsigkas 2022	4	518	8	524	2.8%	0.50 [0.15, 1.68]
Wang 2020	4	312	5	308	1.7%	0.79 [0.21, 2.96]
**Total (95% CI)**		**5435**		**5393**	**100.0%**	**0.67 [0.56, 0.80]**
Total events	225		327			

Heterogeneity: Chi² = 31.15, df = 18 (P = 0.03); I² = 42%
Test for overall effect: Z = 4.31 (P < 0.0001)

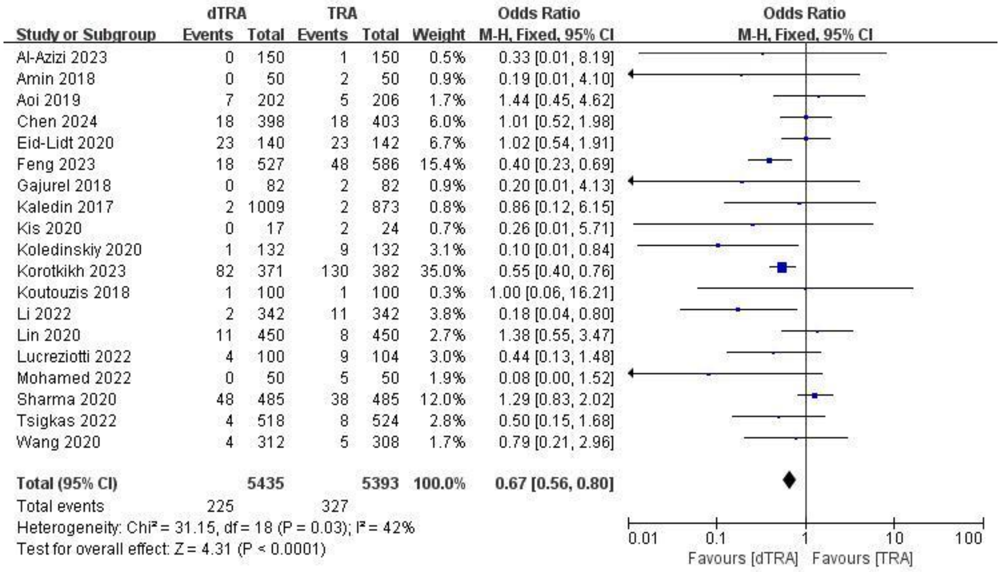

Figure S8. Forest plot of the meta-analysis for radial artery spasm.

Study or Subgroup	dTRA Events	dTRA Total	TRA Events	TRA Total	Weight	Odds Ratio M-H, Random, 95% CI
Aminian 2022	35	650	18	657	10.1%	2.02 [1.13, 3.61]
Aoi 2019	2	202	0	206	2.5%	5.15 [0.25, 107.93]
Chugh 2021	3	209	3	328	5.7%	1.58 [0.32, 7.89]
Eid-Lidt 2020	5	140	6	142	7.3%	0.84 [0.25, 2.82]
Gajurel 2018	2	82	3	82	5.0%	0.66 [0.11, 4.05]
Hammami 2021	5	82	8	95	7.5%	0.71 [0.22, 2.25]
Kis 2020	0	17	1	24	2.2%	0.45 [0.02, 11.66]
Koledinskiy 2020	7	132	17	132	8.6%	0.38 [0.15, 0.95]
Korotkikh 2023	87	371	87	382	11.0%	1.04 [0.74, 1.46]
Koutouzis 2018	3	100	4	100	6.0%	0.74 [0.16, 3.41]
Lalani 2024	2	66	2	64	4.5%	0.97 [0.13, 7.09]
Sharma 2020	5	485	58	485	8.6%	0.08 [0.03, 0.19]
Tsigkas 2022	1	518	0	524	2.3%	3.04 [0.12, 74.81]
Vefalı 2019	0	102	4	103	2.6%	0.11 [0.01, 2.03]
Wang 2020	9	312	12	308	8.8%	0.73 [0.30, 1.76]
Xu 2022	14	151	4	151	7.6%	3.76 [1.21, 11.69]
**Total (95% CI)**		**3619**		**3783**	**100.0%**	**0.80 [0.47, 1.37]**
Total events	180		227			

Heterogeneity: Tau² = 0.65; Chi² = 51.64, df = 15 (P < 0.00001); I² = 71%
Test for overall effect: Z = 0.80 (P = 0.43)

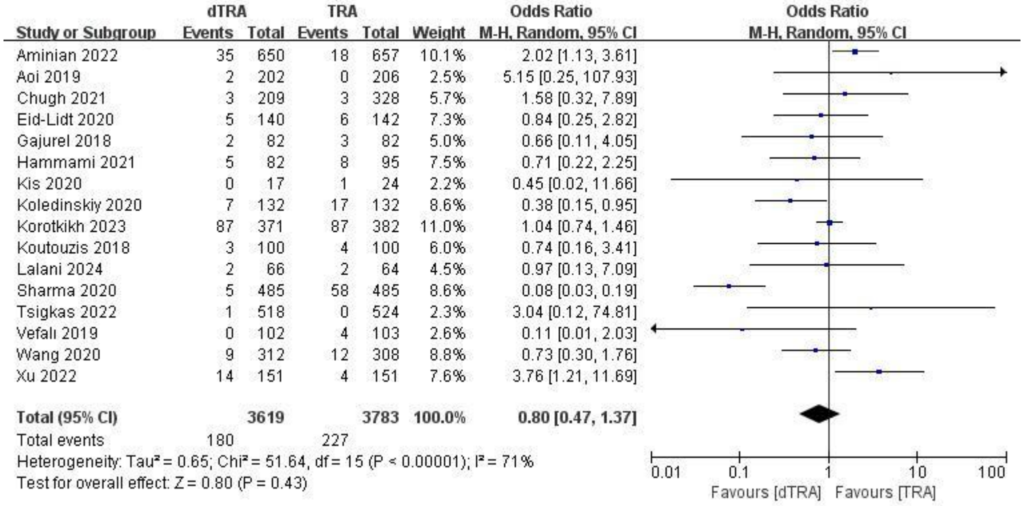

Figure S9. Forest plot of the meta-analysis for puncture time.

	dTRA			TRA				Mean Difference
Study or Subgroup	Mean	SD	Total	Mean	SD	Total	Weight	IV, Random, 95% CI
Amin 2018	1.2	0.94	50	1.2	0.94	50	4.5%	0.00 [-0.37, 0.37]
Aoi 2019	7.3	5.7	202	5.2	4	206	1.6%	2.10 [1.14, 3.06]
Bhambhani 2020	4.1	2.9	100	2.6	2.4	100	2.3%	1.50 [0.76, 2.24]
Chen 2024	1	0.11	382	1	0.02	397	6.5%	0.00 [-0.01, 0.01]
Chugh 2021	2.08	0.9	209	1.17	0.8	328	6.0%	0.91 [0.76, 1.06]
Eid-Lidt 2020	2.7	1.9	140	2.7	2	142	3.9%	0.00 [-0.46, 0.46]
Feng 2023	6.93	7.25	527	3.18	3.52	586	2.6%	3.75 [3.07, 4.43]
Gajurel 2018	1.2	0.94	82	1.2	0.94	82	5.1%	0.00 [-0.29, 0.29]
Hammami 2021	0.7	0.15	82	0.75	0.1	95	6.4%	-0.05 [-0.09, -0.01]
Koledinskiy 2020	2.09	0.2	132	0.98	0.14	132	6.4%	1.11 [1.07, 1.15]
Korotkikh 2023	0.3	0.11	371	0.22	0.06	382	6.5%	0.08 [0.07, 0.09]
Koutouzis 2018	4.48	4.18	100	2.33	2.68	100	1.6%	2.15 [1.18, 3.12]
Lalani 2024	1.29	1.13	66	0.89	0.37	64	5.1%	0.40 [0.11, 0.69]
Li 2022	1.39	0.37	342	1.03	0.07	342	6.4%	0.36 [0.32, 0.40]
Lin 2020	3.9	2.5	450	3.1	2.4	450	4.8%	0.80 [0.48, 1.12]
Lucreziotti 2022	2.28	2.7	100	1.36	1.58	104	2.9%	0.92 [0.31, 1.53]
Mohamed 2022	5.1	1.61	50	2.28	1.16	50	3.2%	2.82 [2.27, 3.37]
Roghani-Dehkordi 2020	4.75	0.46	62	4.65	0.65	53	5.7%	0.10 [-0.11, 0.31]
Tsigkas 2022	2	0.52	518	1.25	0.19	524	6.4%	0.75 [0.70, 0.80]
Vefalı 2019	0.78	0.04	102	0.61	0.08	103	6.5%	0.17 [0.15, 0.19]
Xu 2022	3.47	1.1	151	1.2	0.65	151	5.7%	2.27 [2.07, 2.47]
**Total (95% CI)**			**4218**			**4441**	**100.0%**	**0.72 [0.58, 0.86]**

Heterogeneity: Tau² = 0.08; Chi² = 4379.77, df = 20 (P < 0.00001); I² = 100%
Test for overall effect: Z = 10.06 (P < 0.00001)

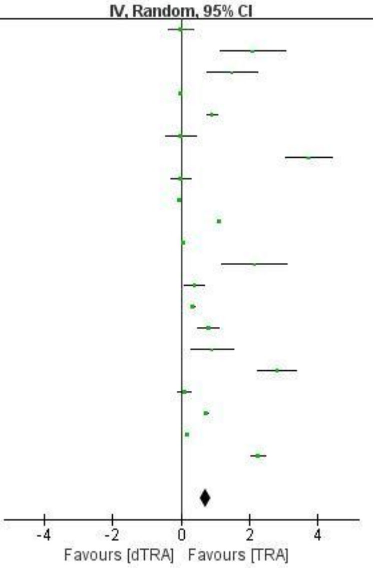

Figure S10. Forest plot of the meta-analysis for procedural time.

	dTRA			TRA				Mean Difference
Study or Subgroup	Mean	SD	Total	Mean	SD	Total	Weight	IV, Random, 95% CI
Aminian 2022	29.1	22.3	627	26.8	20.8	634	8.2%	2.30 [-0.08, 4.68]
Bhambhani 2020	13.4	4.2	100	9.2	3.1	100	9.0%	4.20 [3.18, 5.22]
Chen 2024	25	5.4	398	30	3.97	403	9.1%	-5.00 [-5.66, -4.34]
Coughlan 2018	28.95	5.89	47	29.76	8.16	47	7.8%	-0.81 [-3.69, 2.07]
Korotkikh 2023	20	4.76	371	20	4.43	382	9.1%	0.00 [-0.66, 0.66]
Koutouzis 2018	15.41	14.93	100	13.85	7.06	100	7.5%	1.56 [-1.68, 4.80]
Li 2022	38.5	29.77	342	37.01	29.77	342	6.5%	1.49 [-2.97, 5.95]
Lucreziotti 2022	44.6	28.8	100	46.2	31.4	104	3.8%	-1.60 [-9.86, 6.66]
Mohamed 2022	24	2.91	50	22.28	3.83	50	8.8%	1.72 [0.39, 3.05]
Roghani-Dehkordi 2020	17.5	5.1	62	16.9	4.8	53	8.6%	0.60 [-1.21, 2.41]
Tsigkas 2022	14	2.3	518	11	1.81	524	9.1%	3.00 [2.75, 3.25]
Wang 2020	50	8.3	312	51	7.9	308	8.9%	-1.00 [-2.28, 0.28]
Xu 2022	52.17	31.02	151	70.05	41.77	151	3.8%	-17.88 [-26.18, -9.58]
**Total (95% CI)**			**3178**			**3198**	**100.0%**	**-0.08 [-2.18, 2.01]**

Heterogeneity: Tau² = 12.52; Chi² = 594.41, df = 12 (P < 0.00001); I² = 98%
Test for overall effect: Z = 0.08 (P = 0.94)

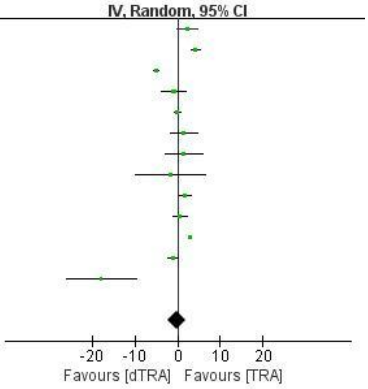

Figure S11. Forest plot of the meta-analysis for dosage of contrast medium.

	dTRA			TRA				Mean Difference
Study or Subgroup	Mean	SD	Total	Mean	SD	Total	Weight	IV, Fixed, 95% CI
Aminian 2022	91.8	65	531	95.2	73.1	525	1.0%	-3.40 [-11.75, 4.95]
Chen 2024	50	6.77	398	50	10.14	403	47.1%	0.00 [-1.19, 1.19]
Coughlan 2018	82.93	23	47	92.1	33	47	0.5%	-9.17 [-20.67, 2.33]
Koutouzis 2018	89	39	100	82	36	100	0.6%	7.00 [-3.40, 17.40]
Li 2022	90.02	74.44	342	91.03	74.44	342	0.5%	-1.01 [-12.17, 10.15]
Lucreziotti 2022	148.2	88.2	100	154.5	92.6	104	0.1%	-6.30 [-31.11, 18.51]
Mohamed 2022	189.4	51.25	50	190	49.16	50	0.2%	-0.60 [-20.28, 19.08]
Roghani-Dehkordi 2020	122	20	62	127	15	53	1.6%	-5.00 [-11.41, 1.41]
Tsigkas 2022	71	11.2	518	70	8.39	524	46.3%	1.00 [-0.20, 2.20]
Vefalı 2019	108.33	22.36	102	107.69	25.54	103	1.6%	0.64 [-5.93, 7.21]
Xu 2022	88.37	53.03	151	101.39	54.33	151	0.5%	-13.02 [-25.13, -0.91]
**Total (95% CI)**			**2401**			**2402**	**100.0%**	**0.28 [-0.54, 1.10]**

Heterogeneity: Chi² = 14.11, df = 10 (P = 0.17); I² = 29%
Test for overall effect: Z = 0.68 (P = 0.50)

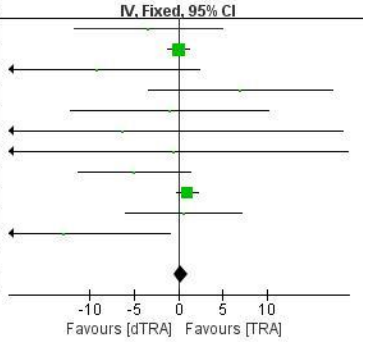

Figure S12. Forest plot of the meta-analysis for hemostasis time.

	dTRA			TRA				Mean Difference
Study or Subgroup	Mean	SD	Total	Mean	SD	Total	Weight	IV, Random, 95% CI
Al-Azizi 2023	0.04	0.01	150	0.04	0.01	150	8.3%	0.00 [-0.00, 0.00]
Aminian 2022	2.79	1.7	581	3.39	1.96	593	7.7%	-0.60 [-0.81, -0.39]
Aoi 2019	1.74	0.67	202	2.97	1.13	206	7.9%	-1.23 [-1.41, -1.05]
Coughlan 2018	2.79	0.5	47	3.94	1.06	47	7.0%	-1.15 [-1.49, -0.81]
Koledinskiy 2020	5.9	0.47	132	2.09	0.26	132	8.2%	3.81 [3.72, 3.90]
Koutouzis 2018	0.15	0.12	100	0.23	0.15	100	8.3%	-0.08 [-0.12, -0.04]
Li 2022	4.35	2.23	342	6.7	1.48	342	7.3%	-2.35 [-2.63, -2.07]
Lin 2020	2.5	0.84	450	3.51	1.01	450	8.1%	-1.01 [-1.13, -0.89]
Lucreziotti 2022	4.15	2.85	100	4.1	2.74	104	4.2%	0.05 [-0.72, 0.82]
Mohamed 2022	0.08	0.01	50	0.4	0.06	50	8.3%	-0.32 [-0.34, -0.30]
Tsigkas 2022	1	0.16	518	2	0.32	524	8.3%	-1.00 [-1.03, -0.97]
Vefalı 2019	0.19	0.03	102	0.33	0.06	103	8.3%	-0.14 [-0.15, -0.13]
Xu 2022	2.88	0.69	151	4.55	0.7	151	8.0%	-1.67 [-1.83, -1.51]
**Total (95% CI)**			**2925**			**2952**	**100.0%**	**-0.43 [-0.65, -0.20]**

Heterogeneity: Tau² = 0.16; Chi² = 13645.70, df = 12 (P < 0.00001); I² = 100%
Test for overall effect: Z = 3.69 (P = 0.0002)

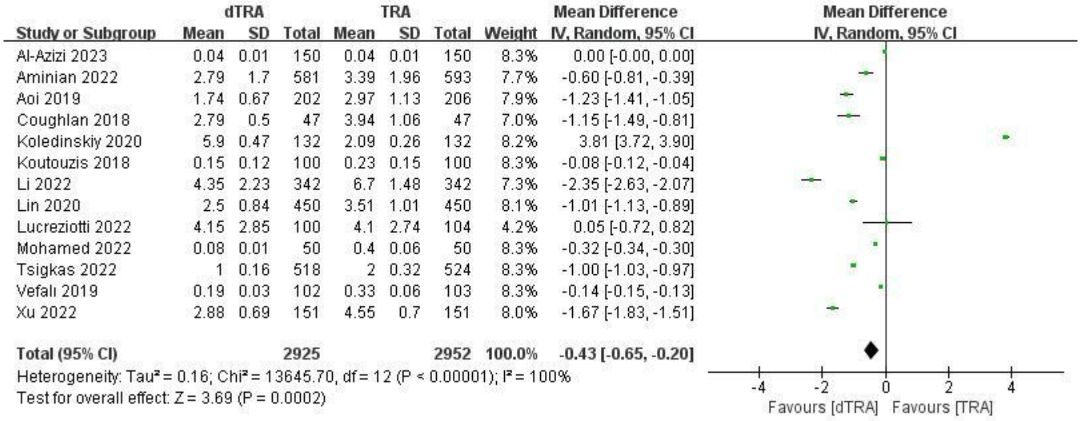

Figure S13.Forest plot of the meta-analysis for RAO.

	dTRA		cTRA			Odds Ratio
Study or Subgroup	Events	Total	Events	Total	Weight	M-H, Fixed, 95% CI
Al-Azizi2023	0	150	1	150	2.1%	0.33 [0.01, 8.19]
Kaledin2017	22	1009	36	873	54.2%	0.52 [0.30, 0.89]
Koledinskiy2020	1	132	8	132	11.4%	0.12 [0.01, 0.96]
Mohamed2022	2	50	7	50	9.6%	0.26 [0.05, 1.30]
Wang2020	6	312	16	308	22.7%	0.36 [0.14, 0.93]
**Total (95% CI)**		**1653**		**1513**	**100.0%**	**0.41 [0.26, 0.63]**
Total events	31		68			

Heterogeneity: Chi² = 2.51, df = 4 (P = 0.64); I² = 0%
Test for overall effect: Z = 4.10 (P < 0.0001)

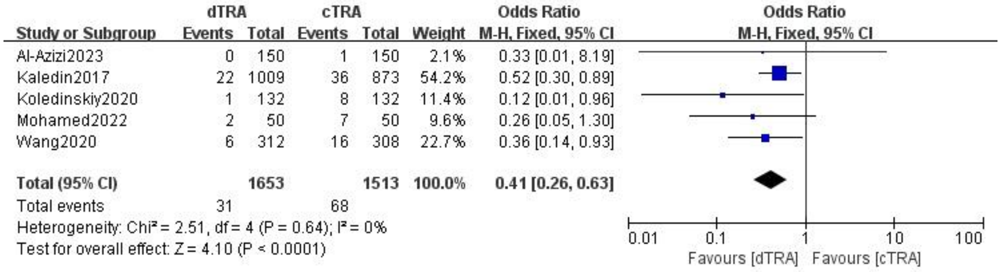

Figure S14. Forest plot of the meta-analysis for puncture point bleeding.

	dTRA		cTRA			Odds Ratio
Study or Subgroup	Events	Total	Events	Total	Weight	M-H, Fixed, 95% CI
Al-Azizi2023	0	150	2	150	17.5%	0.20 [0.01, 4.15]
Li2022	4	123	13	140	82.5%	0.33 [0.10, 1.04]
**Total (95% CI)**		**273**		**290**	**100.0%**	**0.31 [0.10, 0.89]**
Total events	4		15			

Heterogeneity: Chi² = 0.09, df = 1 (P = 0.76); I² = 0%
Test for overall effect: Z = 2.17 (P = 0.03)

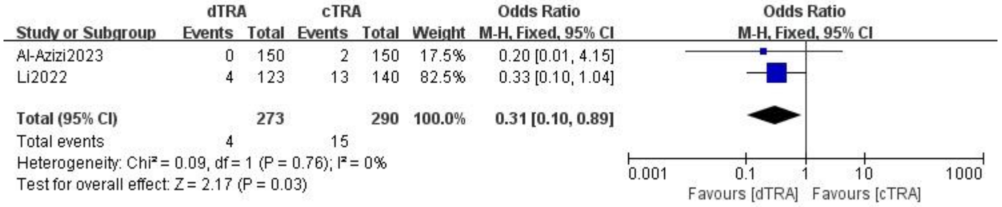

Figures S15. Forest plot of the meta-analysis for success rate of catheter puncture.

	dTRA		cTRA			Odds Ratio
Study or Subgroup	Events	Total	Events	Total	Weight	M-H, Random, 95% CI
Kaledin2017	2704	2775	2975	3099	21.5%	1.59 [1.18, 2.13]
Lalani2024	61	66	63	64	10.4%	0.19 [0.02, 1.71]
Lucreziotti2022	66	100	95	104	19.1%	0.18 [0.08, 0.41]
Mohamed2022	37	50	48	50	14.1%	0.12 [0.03, 0.56]
Vefalı2019	97	102	99	103	15.4%	0.78 [0.20, 3.01]
Wang2020	296	312	295	308	19.4%	0.82 [0.39, 1.72]
**Total (95% CI)**		**3405**		**3728**	**100.0%**	**0.46 [0.18, 1.22]**
Total events	3261		3575			

Heterogeneity: Tau² = 1.11; Chi² = 36.60, df = 5 (P < 0.00001); I² = 86%
Test for overall effect: Z = 1.56 (P = 0.12)

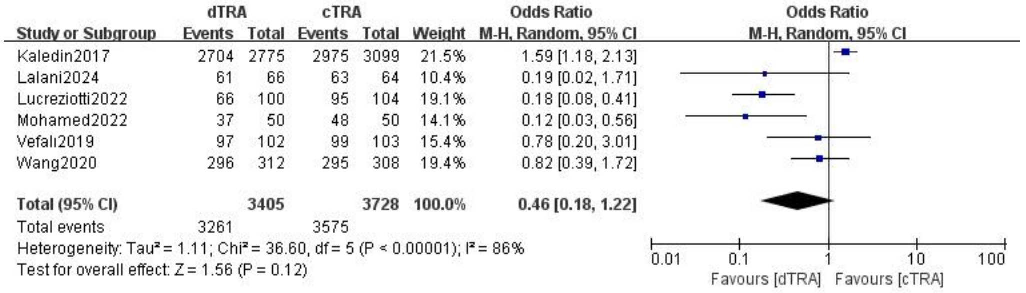

Figure S16. Forest plot of the meta-analysis for hematoma.

Study or Subgroup	dTRA Events	dTRA Total	cTRA Events	cTRA Total	Weight	Odds Ratio M-H, Fixed, 95% CI
Al-Azizi2023	0	150	1	150	4.0%	0.33 [0.01, 8.19]
Kaledin2017	2	1009	2	873	5.8%	0.86 [0.12, 6.15]
Koledinskiy2020	1	132	9	132	24.2%	0.10 [0.01, 0.84]
Li2022	2	123	6	140	14.9%	0.37 [0.07, 1.86]
Lucreziotti2022	4	100	9	104	22.9%	0.44 [0.13, 1.48]
Mohamed2022	0	50	5	50	14.7%	0.08 [0.00, 1.52]
Wang2020	4	312	5	308	13.4%	0.79 [0.21, 2.96]
**Total (95% CI)**		**1876**		**1757**	**100.0%**	**0.36 [0.19, 0.67]**
Total events	13		37			

Heterogeneity: Chi² = 4.54, df = 6 (P = 0.60); I² = 0%
Test for overall effect: Z = 3.21 (P = 0.001)

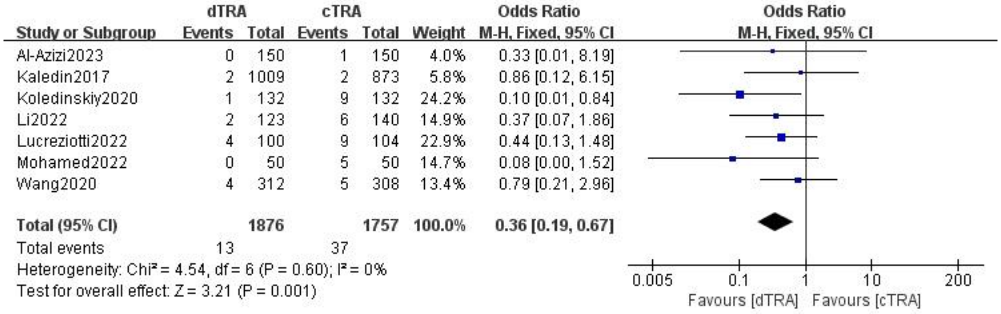

Figure S17. Forest plot of the meta-analysis for radial artery spasm.

Study or Subgroup	dTRA Events	dTRA Total	cTRA Events	cTRA Total	Weight	Odds Ratio M-H, Fixed, 95% CI
Koledinskiy2020	7	132	17	132	47.0%	0.38 [0.15, 0.95]
Lalani2024	2	66	2	64	5.7%	0.97 [0.13, 7.09]
Vefalı2019	0	102	4	103	13.0%	0.11 [0.01, 2.03]
Wang2020	9	312	12	308	34.2%	0.73 [0.30, 1.76]
**Total (95% CI)**		**612**		**607**	**100.0%**	**0.50 [0.28, 0.89]**
Total events	18		35			

Heterogeneity: Chi² = 2.56, df = 3 (P = 0.47); I² = 0%
Test for overall effect: Z = 2.37 (P = 0.02)

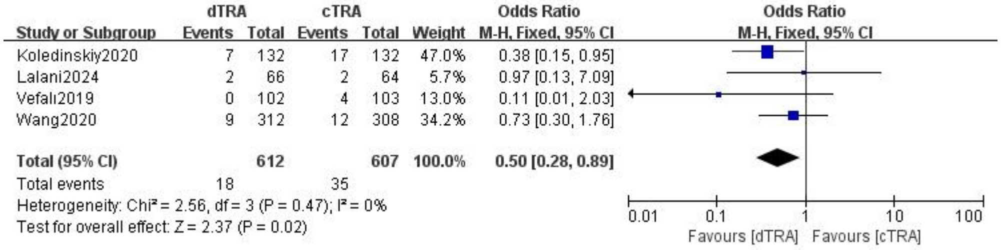

Figure S18. Forest plot of the meta-analysis for puncture time.

Study or Subgroup	dTRA Mean	dTRA SD	dTRA Total	cTRA Mean	cTRA SD	cTRA Total	Weight	Mean Difference IV, Random, 95% CI
Aoi2019	7.1	5.4	90	4.9	3.3	92	9.8%	2.20 [0.90, 3.50]
Koledinskiy2020	2.09	0.2	132	0.98	0.14	132	19.5%	1.11 [1.07, 1.15]
Lalani2024	1.29	1.13	66	0.89	0.37	64	18.6%	0.40 [0.11, 0.69]
Lucreziotti2022	2.28	2.7	100	1.36	1.58	104	16.0%	0.92 [0.31, 1.53]
Mohamed2022	5.1	1.61	50	2.28	1.16	50	16.6%	2.82 [2.27, 3.37]
Vefalı2019	0.78	0.04	102	0.61	0.08	103	19.5%	0.17 [0.15, 0.19]
**Total (95% CI)**			**540**			**545**	**100.0%**	**1.16 [0.57, 1.74]**

Heterogeneity: Tau² = 0.45; Chi² = 1761.57, df = 5 (P < 0.00001); I² = 100%
Test for overall effect: Z = 3.90 (P < 0.0001)

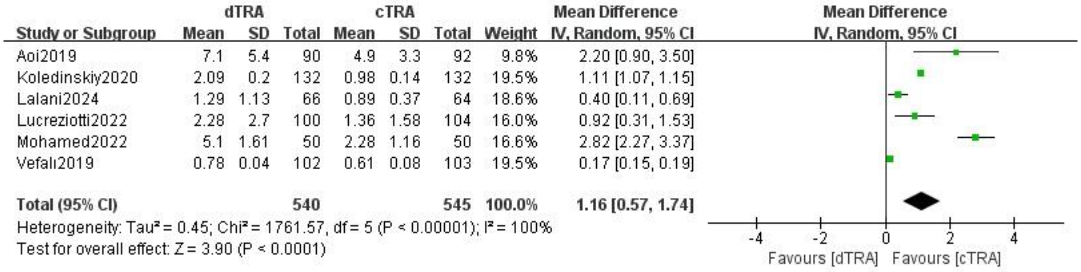

Figure S19. Forest plot of the meta-analysis for procedural time.

Study or Subgroup	dTRA Mean	dTRA SD	dTRA Total	cTRA Mean	cTRA SD	cTRA Total	Weight	Mean Difference IV, Random, 95% CI
Lucreziotti2022	44.6	28.8	100	46.2	31.4	104	7.3%	-1.60 [-9.86, 6.66]
Mohamed2022	24	2.91	50	22.28	3.83	50	46.1%	1.72 [0.39, 3.05]
Wang2020	50	8.3	312	51	7.9	308	46.6%	-1.00 [-2.28, 0.28]
**Total (95% CI)**			**462**			**462**	**100.0%**	**0.21 [-2.19, 2.61]**

Heterogeneity: Tau² = 2.80; Chi² = 8.55, df = 2 (P = 0.01); I² = 77%
Test for overall effect: Z = 0.17 (P = 0.86)

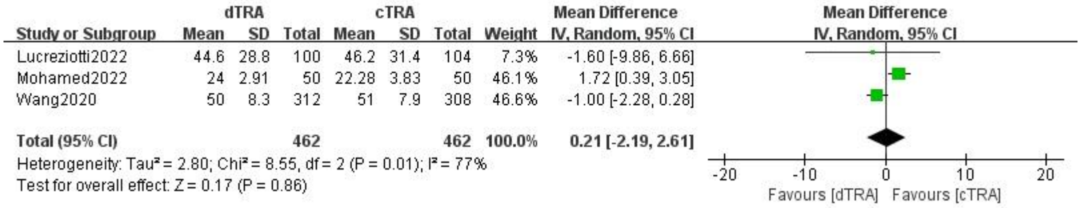

Figure S20. Forest plot of the meta-analysis for dosage of contrast medium.

Study or Subgroup	dTRA Mean	dTRA SD	dTRA Total	cTRA Mean	cTRA SD	cTRA Total	Weight	Mean Difference IV, Fixed, 95% CI
Lucreziotti2022	148.2	88.2	100	154.5	92.6	104	5.9%	-6.30 [-31.11, 18.51]
Mohamed2022	189.4	51.25	50	190	49.16	50	9.4%	-0.60 [-20.28, 19.08]
Vefalı2019	108.33	22.36	102	107.69	25.54	103	84.6%	0.64 [-5.93, 7.21]
**Total (95% CI)**			**252**			**257**	**100.0%**	**0.11 [-5.93, 6.16]**

Heterogeneity: Chi² = 0.29, df = 2 (P = 0.87); I² = 0%
Test for overall effect: Z = 0.04 (P = 0.97)

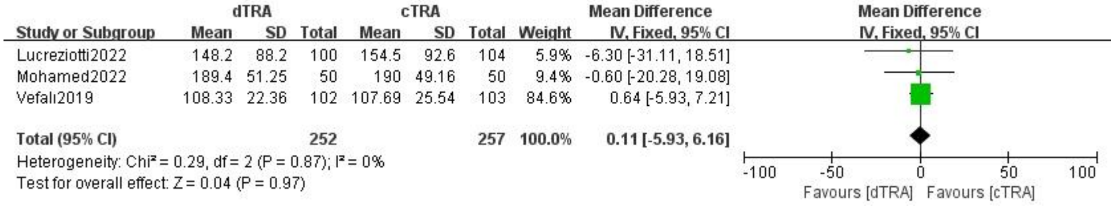

Figure S21. Forest plot of the meta-analysis for hemostasis time.

Study or Subgroup	dTRA Mean	dTRA SD	dTRA Total	cTRA Mean	cTRA SD	cTRA Total	Weight	Mean Difference IV, Random, 95% CI
Al-Azizi2023	0.04	0.01	150	0.04	0.01	150	18.7%	0.00 [-0.00, 0.00]
Aoi2019	2.01	0.75	90	4.09	0.65	92	17.2%	-2.08 [-2.28, -1.88]
Koledinskiy2020	5.9	0.47	132	2.09	0.26	132	18.4%	3.81 [3.72, 3.90]
Lucreziotti2022	4.15	2.85	100	4.1	2.74	104	8.4%	0.05 [-0.72, 0.82]
Mohamed2022	0.08	0.01	50	0.4	0.06	50	18.7%	-0.32 [-0.34, -0.30]
Vefalı2019	0.19	0.03	102	0.33	0.06	103	18.7%	-0.14 [-0.15, -0.13]
**Total (95% CI)**			**624**			**631**	**100.0%**	**0.26 [-0.04, 0.56]**

Heterogeneity: Tau² = 0.12; Chi² = 8826.95, df = 5 (P < 0.00001); I² = 100%
Test for overall effect: Z = 1.71 (P = 0.09)

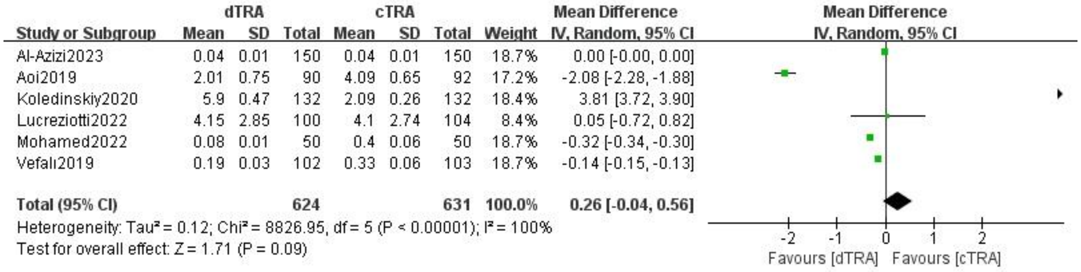

Figure S22. Forest plot of the meta-analysis for RAO.

Study or Subgroup	dTRA Events	dTRA Total	cTRA Events	cTRA Total	Weight	Odds Ratio M-H, Fixed, 95% CI
Al-Azizi2023	0	150	2	150	1.3%	0.20 [0.01, 4.15]
Aminian2022	2	650	6	657	3.2%	0.33 [0.07, 1.67]
Chen2024	10	398	27	403	14.1%	0.36 [0.17, 0.75]
Eid-Lidt2020	2	140	8	142	4.2%	0.24 [0.05, 1.16]
Koledinskiy2020	1	132	8	132	4.3%	0.12 [0.01, 0.96]
Korotkikh 2023	15	371	26	382	13.3%	0.58 [0.30, 1.11]
Koutouzis2018	5	100	9	100	4.6%	0.53 [0.17, 1.65]
Mohamed2022	2	50	7	50	3.6%	0.26 [0.05, 1.30]
Mokbel2018	0	57	3	57	1.9%	0.14 [0.01, 2.68]
Sharma2020	10	485	63	485	33.3%	0.14 [0.07, 0.28]
Tsigkas2022	19	518	31	524	16.0%	0.61 [0.34, 1.09]
**Total (95% CI)**		**3051**		**3082**	**100.0%**	**0.34 [0.25, 0.45]**
Total events	66		190			

Heterogeneity: Chi² = 15.16, df = 10 (P = 0.13); I² = 34%
Test for overall effect: Z = 7.52 (P < 0.00001)

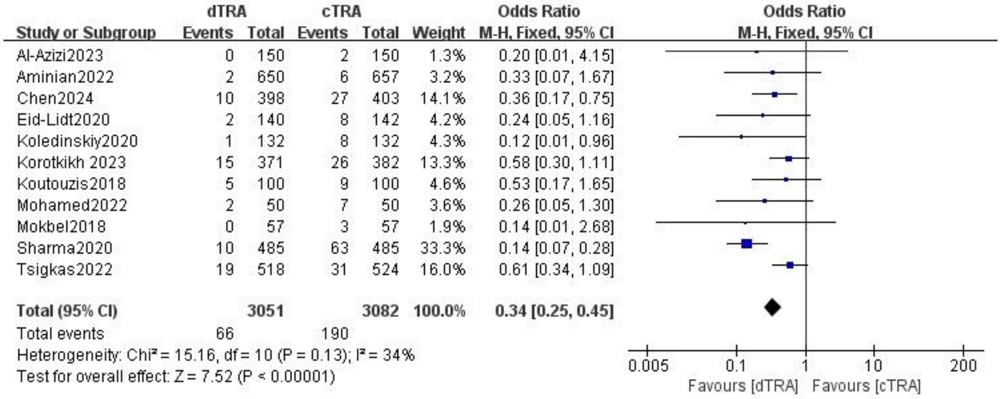

Figure S23. Forest plot of the meta-analysis for success rate of catheter puncture.

Study or Subgroup	dTRA Events	dTRA Total	cTRA Events	cTRA Total	Weight	Odds Ratio M-H, Random, 95% CI
Aminian2022	525	650	528	656	20.2%	1.02 [0.77, 1.34]
Chen2024	382	398	397	403	16.5%	0.36 [0.14, 0.93]
Lucreziotti2022	66	100	95	104	17.5%	0.18 [0.08, 0.41]
Mohamed2022	37	50	48	50	12.5%	0.12 [0.03, 0.56]
Tsigkas2022	408	518	497	524	19.5%	0.20 [0.13, 0.31]
Vefalı2019	97	102	99	103	13.8%	0.78 [0.20, 3.01]
**Total (95% CI)**		**1818**		**1840**	**100.0%**	**0.34 [0.14, 0.82]**
Total events	1515		1664			

Heterogeneity: Tau² = 0.96; Chi² = 51.71, df = 5 (P < 0.00001); I² = 90%
Test for overall effect: Z = 2.42 (P = 0.02)

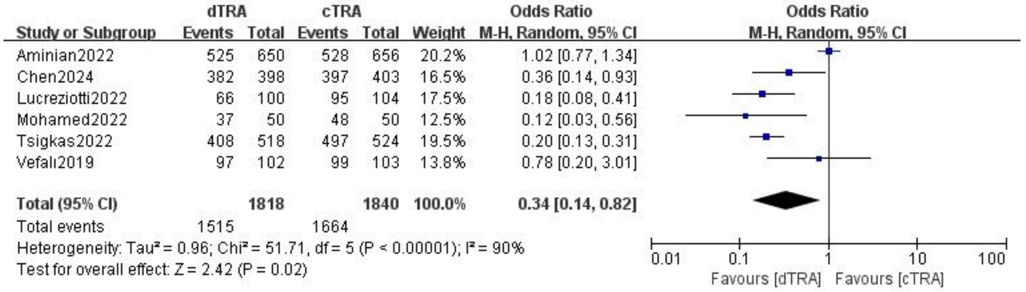

Figure S24. Forest plot of the meta-analysis for success rate of a single attempt.

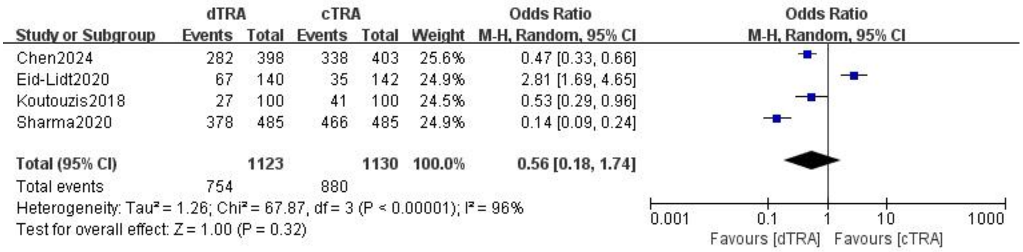

Figure S25. Forest plot of the meta-analysis for puncture point bleeding.

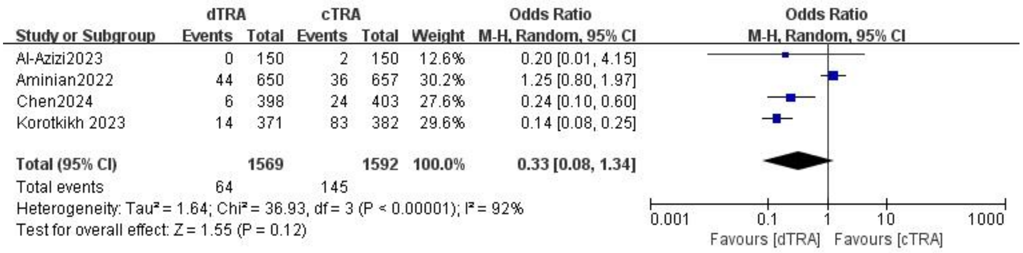

Figure S26. Forest plot of the meta-analysis for procedure success.

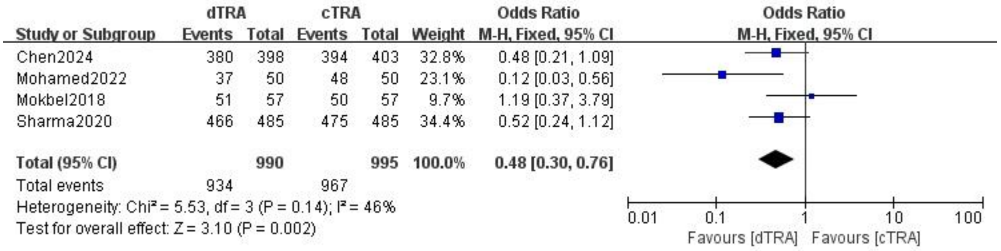

Figure S27. Forest plot of the meta-analysis for hematoma.

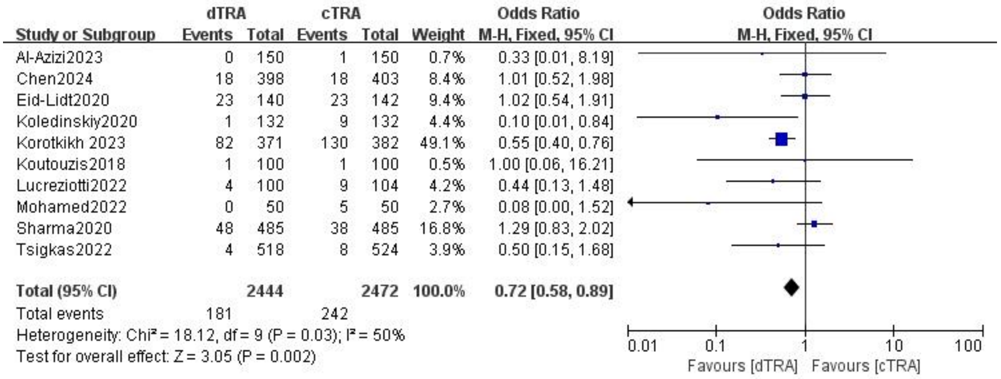

Figure S28. Forest plot of the meta-analysis for radial artery spasm.

Study or Subgroup	dTRA Events	dTRA Total	cTRA Events	cTRA Total	Weight	Odds Ratio M-H, Random, 95% CI
Aminian2022	35	650	18	657	16.9%	2.02 [1.13, 3.61]
Eid-Lidt2020	5	140	6	142	13.3%	0.84 [0.25, 2.82]
Koledinskiy2020	7	132	17	132	15.0%	0.38 [0.15, 0.95]
Korotkikh 2023	87	371	87	382	17.8%	1.04 [0.74, 1.46]
Koutouzis2018	3	100	4	100	11.4%	0.74 [0.16, 3.41]
Sharma2020	5	485	58	485	15.0%	0.08 [0.03, 0.19]
Tsigkas2022	1	518	0	524	5.0%	3.04 [0.12, 74.81]
Vefalı2019	0	102	4	103	5.6%	0.11 [0.01, 2.03]
**Total (95% CI)**		**2498**		**2525**	**100.0%**	**0.59 [0.25, 1.35]**
Total events	143		194			

Heterogeneity: Tau² = 0.99; Chi² = 43.70, df = 7 (P < 0.00001); I² = 84%
Test for overall effect: Z = 1.25 (P = 0.21)

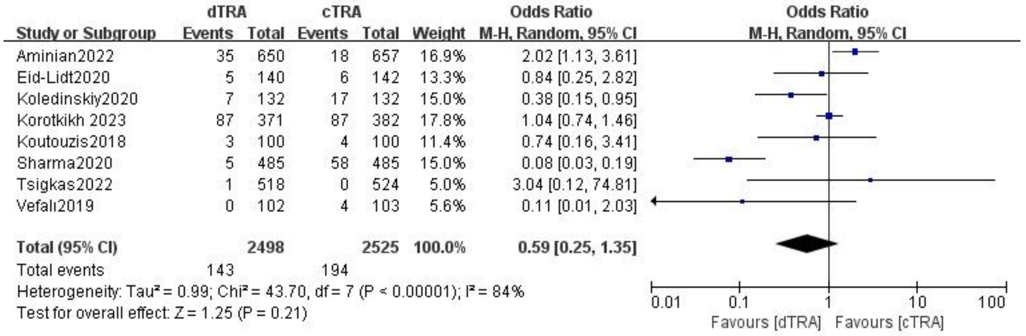

Figure S29. Forest plot of the meta-analysis for puncture time.

Study or Subgroup	dTRA Mean	dTRA SD	dTRA Total	cTRA Mean	cTRA SD	cTRA Total	Weight	Mean Difference IV, Random, 95% CI
Chen2024	1	0.11	382	1	0.02	397	14.9%	0.00 [-0.01, 0.01]
Eid-Lidt2020	2.7	1.9	140	2.7	2	142	8.6%	0.00 [-0.46, 0.46]
Koledinskiy2020	2.09	0.2	132	0.98	0.14	132	14.9%	1.11 [1.07, 1.15]
Korotkikh 2023	0.3	0.11	371	0.22	0.06	382	14.9%	0.08 [0.07, 0.09]
Koutouzis2018	4.48	4.18	100	2.33	2.68	100	3.4%	2.15 [1.18, 3.12]
Lucreziotti2022	2.28	2.7	100	1.36	1.58	104	6.4%	0.92 [0.31, 1.53]
Mohamed2022	5.1	1.61	50	2.28	1.16	50	7.2%	2.82 [2.27, 3.37]
Tsigkas2022	2	0.52	518	1.25	0.19	524	14.8%	0.75 [0.70, 0.80]
Vefalı2019	0.78	0.04	102	0.61	0.08	103	14.9%	0.17 [0.15, 0.19]
**Total (95% CI)**			**1895**			**1934**	**100.0%**	**0.65 [0.44, 0.85]**

Heterogeneity: Tau² = 0.07; Chi² = 3462.94, df = 8 (P < 0.00001); I² = 100%
Test for overall effect: Z = 6.22 (P < 0.00001)

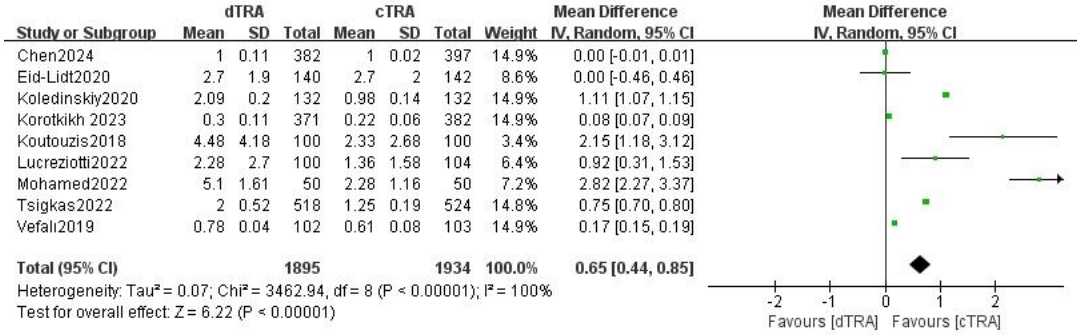

Figure S30. Forest plot of the meta-analysis for procedural time.

Study or Subgroup	dTRA Mean	dTRA SD	dTRA Total	cTRA Mean	cTRA SD	cTRA Total	Weight	Mean Difference IV, Random, 95% CI
Aminian2022	29.1	22.3	627	26.8	20.8	634	14.8%	2.30 [-0.08, 4.68]
Chen2024	25	5.4	398	30	3.97	403	16.1%	-5.00 [-5.66, -4.34]
Korotkikh 2023	20	4.76	371	20	4.43	382	16.1%	0.00 [-0.66, 0.66]
Koutouzis2018	15.41	14.93	100	13.85	7.06	100	13.7%	1.56 [-1.68, 4.80]
Lucreziotti2022	44.6	28.8	100	46.2	31.4	104	7.4%	-1.60 [-9.86, 6.66]
Mohamed2022	24	2.91	50	22.28	3.83	50	15.7%	1.72 [0.39, 3.05]
Tsigkas2022	14	2.3	518	11	1.81	524	16.2%	3.00 [2.75, 3.25]
**Total (95% CI)**			**2164**			**2197**	**100.0%**	**0.39 [-2.68, 3.45]**

Heterogeneity: Tau² = 15.06; Chi² = 528.44, df = 6 (P < 0.00001); I² = 99%
Test for overall effect: Z = 0.25 (P = 0.80)

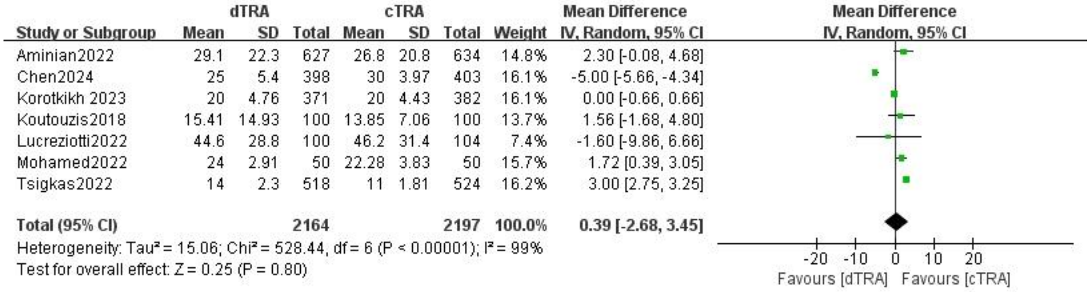

Figure S31. Forest plot of the meta-analysis for dosage of contrast medium.

Study or Subgroup	dTRA Mean	dTRA SD	dTRA Total	cTRA Mean	cTRA SD	cTRA Total	Weight	Mean Difference IV, Fixed, 95% CI
Aminian2022	91.8	65	531	95.2	73.1	525	1.0%	-3.40 [-11.75, 4.95]
Chen2024	50	6.77	398	50	10.14	403	48.6%	0.00 [-1.19, 1.19]
Koutouzis2018	89	39	100	82	36	100	0.6%	7.00 [-3.40, 17.40]
Lucreziotti2022	148.2	88.2	100	154.5	92.6	104	0.1%	-6.30 [-31.11, 18.51]
Mohamed2022	189.4	51.25	50	190	49.16	50	0.2%	-0.60 [-20.28, 19.08]
Tsigkas2022	71	11.2	518	70	8.39	524	47.8%	1.00 [-0.20, 2.20]
Vefalı2019	108.33	22.36	102	107.69	25.54	103	1.6%	0.64 [-5.93, 7.21]
**Total (95% CI)**			**1799**			**1809**	**100.0%**	**0.49 [-0.34, 1.32]**

Heterogeneity: Chi² = 3.98, df = 6 (P = 0.68); I² = 0%
Test for overall effect: Z = 1.16 (P = 0.25)

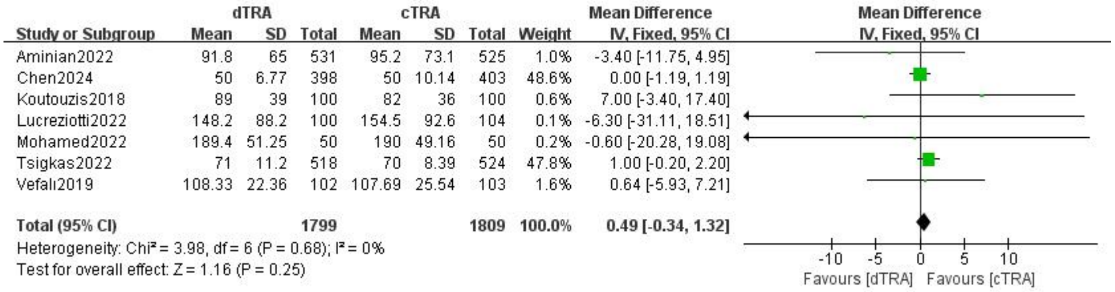

Figure S32. Forest plot of the meta-analysis for hemostasis time.

Study or Subgroup	dTRA Mean	dTRA SD	dTRA Total	cTRA Mean	cTRA SD	cTRA Total	Weight	Mean Difference IV, Random, 95% CI
Al-Azizi2023	0.04	0.01	150	0.04	0.01	150	13.5%	0.00 [-0.00, 0.00]
Aminian2022	2.79	1.7	581	3.39	1.96	593	12.5%	-0.60 [-0.81, -0.39]
Koledinskiy2020	5.9	0.47	132	2.09	0.26	132	13.3%	3.81 [3.72, 3.90]
Koutouzis2018	0.15	0.12	100	0.23	0.15	100	13.5%	-0.08 [-0.12, -0.04]
Lucreziotti2022	4.15	2.85	100	4.1	2.74	104	6.6%	0.05 [-0.72, 0.82]
Mohamed2022	0.08	0.01	50	0.4	0.06	50	13.5%	-0.32 [-0.34, -0.30]
Tsigkas2022	1	0.16	518	2	0.32	524	13.5%	-1.00 [-1.03, -0.97]
Vefalı2019	0.19	0.03	102	0.33	0.06	103	13.5%	-0.14 [-0.15, -0.13]
**Total (95% CI)**			**1733**			**1756**	**100.0%**	**0.23 [-0.05, 0.51]**

Heterogeneity: $Tau^2 = 0.15$; $Chi^2 = 12476.94$, df = 7 ($P < 0.00001$); $I^2 = 100\%$

Test for overall effect: Z = 1.61 (P = 0.11)

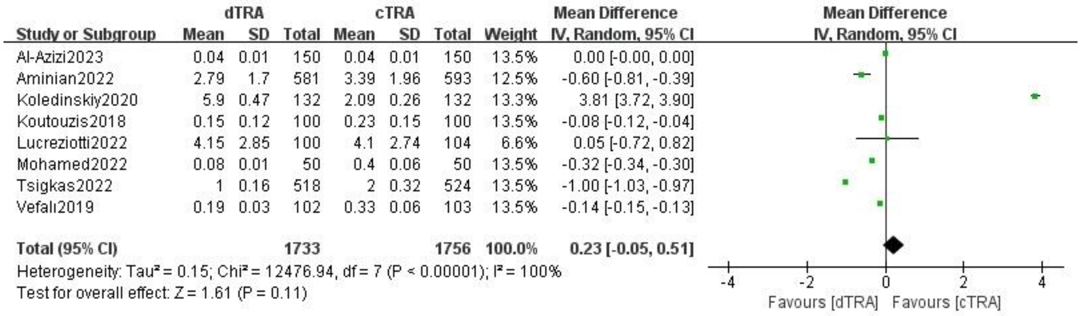

Supplement: Supplementary file 2 [file Datasheet2.pdf]
